# Multiomics integration of 22 immune-mediated monogenic diseases reveals an emergent axis of human immune health

**DOI:** 10.21203/rs.3.rs-2070975/v1

**Published:** 2023-03-20

**Authors:** Rachel Sparks, Nicholas Rachmaninoff, Dylan C. Hirsch, Neha Bansal, William W. Lau, Andrew J. Martins, Jinguo Chen, Candace C. Liu, Foo Cheung, Laura E. Failla, Angelique Biancotto, Giovanna Fantoni, Brian A. Sellers, Daniel G. Chawla, Katherine N. Howe, Darius Mostaghimi, Rohit Farmer, Yuri Kotliarov, Katherine R. Calvo, Cindy Palmer, Janine Daub, Ladan Foruraghi, Samantha Kreuzburg, Jennifer Treat, Amanda K. Urban, Anne Jones, Tina Romeo, Natalie T. Deuitch, Natalia Sampaio Moura, Barbara Weinstein, Susan Moir, Luigi Ferrucci, Karyl S. Barron, Ivona Aksentijevich, Steven H. Kleinstein, Danielle M. Townsley, Neal S. Young, Pamela A. Frischmeyer-Guerrerio, Gulbu Uzel, Gineth Paola Pinto-Patarroyo, Cornelia D. Cudrici, Patrycja Hoffmann, Deborah L. Stone, Amanda K. Ombrello, Alexandra F. Freeman, Christa S. Zerbe, Daniel L. Kastner, Steven M. Holland, John S. Tsang

**Affiliations:** 1 Multiscale Systems Biology Section, Laboratory of Immune System Biology, NIAID, NIH, Bethesda, MD 20892, USA; 2 Graduate Program in Biological Sciences, University of Maryland, College Park, MD 20742, USA; 3 Office of Intramural Research, CIT, NIH, Bethesda, MD 20892, USA; 4 NIH Center for Human Immunology, NIAID, NIH, Bethesda, MD 20892, USA; 5 Interdepartmental Program in Computational Biology and Bioinformatics, Yale University, New Haven, CT 06511, USA; 6 Laboratory of Clinical Immunology and Microbiology, NIAID, NIH, Bethesda, MD 20892, USA; 7 Hematology Section, Department of Laboratory Medicine, NIH Clinical Center, Bethesda, MD 20892, USA; 8 Clinical Research Directorate, Frederick National Laboratory for Cancer Research, National Cancer Institute, NIH, Frederick, MD 21701, USA.; 9 Inflammatory Diseases Section, National Human Genome Research Institute, NIH, Bethesda, MD 20892, USA; 10 Hematology Branch, National Heart, Lung, and Blood Institute, NIH, Bethesda, MD 20892, USA; 11 Translational Gerontology Branch, National Institute on Aging, Baltimore, MD 21224, USA; 12 Divison of Intramural Research, NIAID, NIH, Bethesda, MD 20892, USA; 13 Department of Immunobiology, Yale University School of Medicine, New Haven, CT 06510, USA; 14 Department of Pathology, Yale University School of Medicine, New Haven, CT 06510, USA; 15 Laboratory of Immunoregulation, NIAID, NIH, Bethesda, MD 20892, USA; 16 Laboratory of Allergic Diseases, NIAID, NIH, Bethesda, MD 20892, USA; 17 National Institute of Arthritis and Musculoskeletal and Skin Diseases, NIH, Bethesda MD 20892, USA

## Abstract

Monogenic diseases are often studied in isolation due to their rarity. Here we utilize multiomics to assess 22 monogenic immune-mediated conditions with age- and sex-matched healthy controls. Despite clearly detectable disease-specific and “pan-disease” signatures, individuals possess stable personal immune states over time. Temporally stable differences among subjects tend to dominate over differences attributable to disease conditions or medication use. Unsupervised principal variation analysis of personal immune states and machine learning classification distinguishing between healthy controls and patients converge to a metric of immune health (IHM). The IHM discriminates healthy from multiple polygenic autoimmune and inflammatory disease states in independent cohorts, marks healthy aging, and is a pre-vaccination predictor of antibody responses to influenza vaccination in the elderly. We identified easy-to-measure circulating protein biomarker surrogates of the IHM that capture immune health variations beyond age. Our work provides a conceptual framework and biomarkers for defining and measuring human immune health.

## Introduction

Immune system dysregulation is central to diverse pathologies, including cancer, chronic inflammation, cardiovascular, and neurological diseases^1^. Immune-mediated disease results from a complex interplay of environmental, exposure history, and genetic factors. In contrast to polygenic diseases such as rheumatoid arthritis (RA) and systemic lupus erythematosus (SLE), monogenic diseases offer unique opportunities to highlight important mechanisms by which individual genes and associated pathways contribute to immune function in humans. For example, the study of patients with immunodeficiencies has illuminated the critical roles of the JAK-STAT network in orchestrating microbial defense and inflammatory processes at the organismal level in humans^2,3^; similarly, monogenic periodic fever syndromes have deepened our molecular understanding of inflammasomes and their roles in innate immunity and autoinflammatory diseases^4^.

Aside from comparison of genetic associations and gene expression quantitative trait loci in polygenic diseases^5-8^, immune-mediated diseases, in particular those of monogenic origin, have often been studied in isolation. Molecular and cellular attributes and biomarkers shared across diseases remained poorly defined, knowledge of which could help advance our understanding of both common and disease-specific pathophysiology and immune dysregulation, potentially pointing to multi-disease therapeutic targets. Importantly, the contribution of genetics to human immune variations can be highly variable and tends in wane by adulthood^9^; even monogenic disease patients with primary causal defects in the same gene can exhibit extensive clinical heterogeneity^10^ with poorly understood molecular and cellular drivers. Thus, dissecting the inter- and intra-patient variations in diverse immune parameters both within and across diseases is critical to understanding disease- and patient-specific dysregulation beyond the causal gene and proximal pathways. Analyzing diverse monogenic diseases may also simultaneously reveal features of a normal, healthy immune system, which remains ill-defined because parameters quantifying immunological health remain elusive^11^. In principle, immune health metrics should not be defined based on features of the immune systems among healthy individuals alone, but also incorporate common features of immune pathologies as “negative” indicators of health. Simultaneous assessment of immune states in monogenic disease patients and matching healthy subjects may thus reveal quantifiable parameters of human immune health.

Here we have integrated multiomics profiling and clinical information to comparatively analyze 22 monogenic immune-mediated disease cohorts together with age- and sex-matched healthy controls. Using this new dataset, we identified both disease-specific and shared (“pan-disease”) signatures, and importantly, found that both patients and healthy subjects possessed temporally stable personal immune states independent of disease condition or medication use^12-14^. Integration of transcriptomic, serum protein, and peripheral blood cell frequency data revealed a quantitative metric of immune health through both bottom-up, unsupervised principal variation analysis of personal immune states and supervised machine learning analyses that discriminated between healthy individuals and sick patients. This metric also marks healthy aging and is associated with the antibody responses to influenza vaccination in the elderly. We also uncovered easy-to-measure serum protein surrogates of this metric that capture immune health variations among healthy individuals beyond age. Beyond our specific findings, this rich dataset can serve as a resource for the research community to probe these specific monogenic disorders more deeply, for example, by generating new hypotheses. Our work paves the way for a more quantitative understanding of human immune health and provides a unique dataset for further exploration.

## Results

### A multiomics compendium of 22 monogenic immune-mediated diseases reveals temporally stable individual differences tend to be the dominant source of variation

**Extended Data Figure 1.**
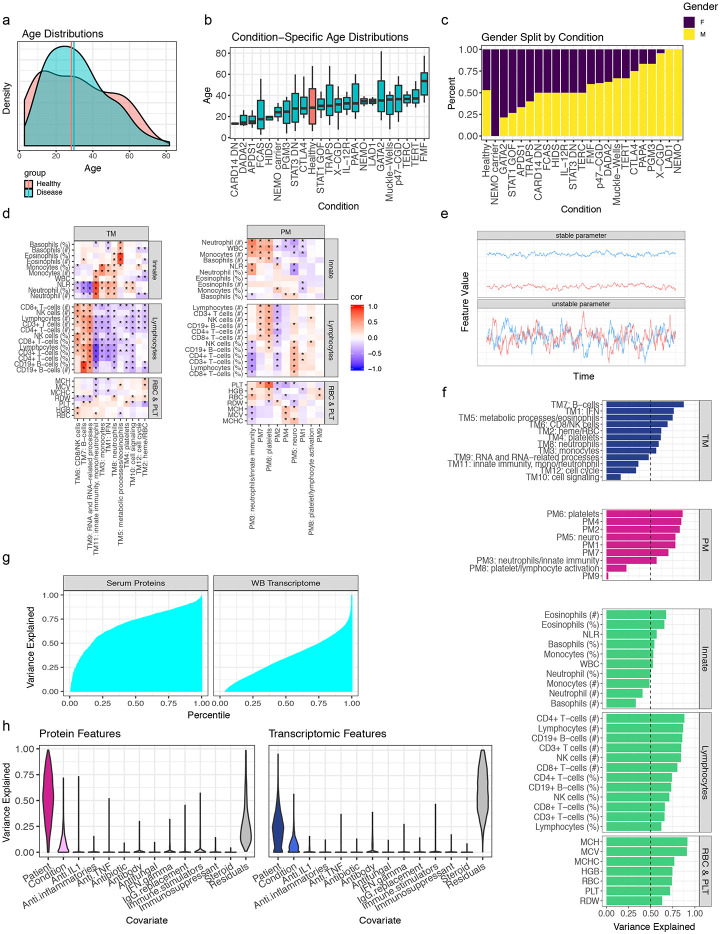
Subject demographics and further characterization of the serum protein and transcriptomic modules. **a,** Density plot of patient and healthy subjects’ age distributions (Kolmogorov-Smirnov test assessing difference between the two distributions, *p* = 0.41). Extended Data Fig. 1a-c only show data for subjects in primary set of subjects; data for set-aside subjects not shown but included in Table 1. **b,** Boxplots of subject ages in each subject group with healthy in red. Box plot center lines correspond to the median value; lower and upper hinges correspond to the first and third quartiles (the 25th and 75th percentiles), and lower and upper whiskers extend from the box to the smallest or largest value correspondingly, but no further than 1.5X inter-quantile range. **c,** Barplots depicting sex distribution within each group shown as male/female proportions. **d,** Pearson correlation between the protein (left) or transcriptomic (right) WGCNA modules (columns) and cellular [complete blood count (CBC) and lymphocyte (T, B, NK cell) phenotyping (TBNK)] parameters (rows). *adjusted *p* value < 0.05. Computed with 198 subjects with both whole blood transcriptome and CBC/TBNK data, and 197 subjects with both serum protein and CBC/TBNK data. TM = whole blood transcriptomic modules. PM = serum protein modules. IFN = interferon. NLR = neutrophil-to-lymphocyte ratio. WBC = white blood cell count. MCHC = mean corpuscular hemoglobin concentration. HGB = hemoglobin. RDW = red cell distribution width. PLT = platelet count. MCH = mean corpuscular hemoglobin. MCV = mean corpuscular volume. RBC = red blood cell count. NK = natural killer. **e,** Conceptual illustration of parameter temporal stability, defined by low intra-subject variation relative to inter-subject variation. **f,** Barplots of variance assigned to the subject term in the variance partition analysis fit using only a subject random intercept (see Methods), run across each CBC parameter, protein module, and transcriptomic module. TM = whole blood transcriptomic modules. PM = serum protein modules. RBC = red blood cell parameters. PLT = platelets. **g,** Percent variation explained by the subject term in the variance partition model in the protein and transcriptomic features using the variance partition model with only a subject random intercept (see Methods) as in (**f**). Proteins (left) and genes (right) are ordered on the x-axis by the percent variation explained by the subject term. WB = whole blood. **h,** Percent variation explained by the patient and medication covariate (showing effect of each medication individually) for each protein (left) and gene (right) measured. Medications were included in the model if they were used by many patients and not highly confounded with one of the condition groups.

**Figure 1.**
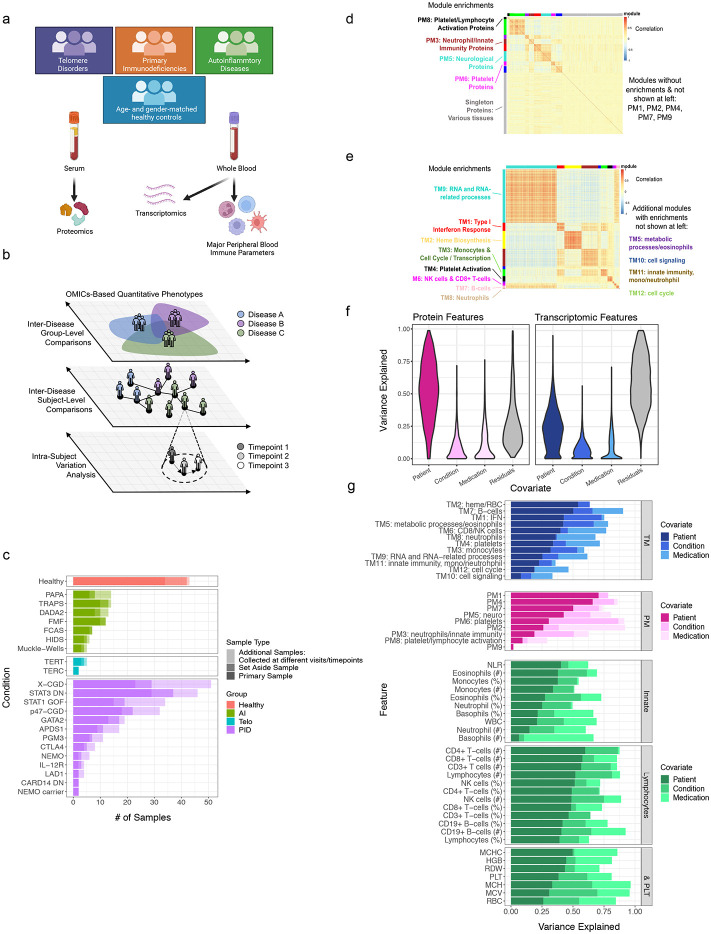
Study and data overview. **a**, Patient groups and data collected. Individual disease groups are shown in (**c**). **b,** Conceptual overview of the study and analysis approaches. Both disease group centric (top-down, disease label based) and individual subject based (bottom-up, unbiasedly starting from subject-subject similarities) analyses are pursued. **c,** Breakdown of cohort by disease and sample type. Data are broken down into the number of “primary” samples (equal to the number of subjects analyzed in this study), subjects reserved (“set aside”) up front immediately after data generation and before any data analyses for potential independent follow-up analyses (see Methods), and samples from the primary subjects (“repeat”) but collected at additional timepoints. AI = autoinflammatory diseases. Telo = telomere disorders. PID = primary immunodeficiencies. **d,** Gene-gene correlation heatmap of whole blood transcriptomic data. Modules of correlated genes [or “transcriptional modules” (TMs); k = 12] are annotated by color at the top and left. Modules were created using all transcriptional features; however, only the temporally stable genes are shown in the heatmap (see (**f**) and (**g**) below). Only modules with significant enrichments are labeled/annotated. **e,** Similar to (**d**) but for serum protein data. Modules of correlated proteins (PMs; k = 10) are annotated by color at the top and left. The serum protein data contains a large, weakly correlated set of proteins (grey module). Modules were created using all features; however, only the temporally stable proteins are shown in the heatmap [see (**f**) and (**g**) below]. Only modules with significant enrichments are labeled/annotated. **f,** Violin plots showing the distribution, across all measured proteins (1,305) and transcripts (15,729), of the percent of variance assigned to each variable in the variance partition analysis. The transcriptomic data had 276 samples with 62 subjects with repeated sampling. The serum protein data consisted of 271 samples with 64 subjects with repeated sampling. **g,** Barplots of the percent of variance assigned to each variable in the variance partition analysis, run across each transcriptomic module (blue), serum protein module (magenta), and CBC parameter (green). This analysis used subjects with repeat samples collected at different timepoints. The CBC/TBNK data consisted of 271 samples with 63 subjects with repeated sampling. TM = whole blood transcriptomic modules. PM = serum protein modules. IFN = interferon. NLR = neutrophil-to-lymphocyte ratio. WBC = white blood cell count. MCHC = mean corpuscular hemoglobin concentration. HGB = hemoglobin. RDW = red cell distribution width. PLT = platelet count. MCH = mean corpuscular hemoglobin. MCV = mean corpuscular volume. RBC = red blood cell count. NK = natural killer.

We employed multiomics analyses of circulating immune cells involving whole blood transcriptomics, measurements of more than 1300 circulating proteins from serum (using the Somalogic platform), as well as immune cell frequencies and hematological parameters from a complete blood count (CBC) and clinical flow cytometry [TBNK: CD4+ and CD8+ T-cells, B-cells, natural killer (NK) cells] to comparatively analyze samples collected from 364 visits of 228 patients (some patients had multiple samples collected at different visits/timepoints)—spanning 22 monogenic immune-mediated diseases—and 42 age- and sex-matched healthy subjects (Fig. 1a-c, Extended Data Fig. 1a-c, Table 1, Extended Data Table 1). Once data were generated, we set aside a set of subjects including patients from the majority of disease groups and matched healthy controls (see Table 1) to enable potential future independent validation or follow-up analyses (see Methods). This monogenic disease compendium includes primary immunodeficiencies, autoinflammatory disorders, and defects in hematopoiesis, each with known causal genetic mutations affecting major molecular and cellular networks and functions of the innate [e.g., NOD-, LRR- and pyrin domain-containing protein 3 (NLRP3)] and adaptive [e.g., signal transducer and activator of transcription 1 (STAT1)] immune systems. Disease manifestations cover a spectrum of features including frequent and severe infections, autoimmunity, allergy, and recurrent fever with inflammation (autoinflammation). Thus, this multi-disease cohort offers unique opportunities for examining the shared and distinct features of these natural genetic perturbations in humans at the molecular and cellular levels. To the best of our knowledge, this constitutes the first and largest multiomics/multimodal comparative map of diverse monogenic, immune-mediated diseases in humans.

To reduce data dimensionality and assess the correlation among parameters, weighted gene correlation network analysis (WGNCA)^15^ was applied to the serum protein and transcriptomic data to derive co-expression modules separately for each data modality. This resulted in 12 blood transcriptomic modules (TMs; Fig. 1d, Extended Data Table 2) and 10 protein modules (PMs; Fig. 1e, Extended Data Table 3). Most of the TMs were enriched for signatures of major immune cell types (e.g., B-cells in TM7; Extended Data Table 4, Extended Data Fig. 1d) or intracellular processes (Extended Data Table 4). A subset of the proteins also formed modules based on co-expression (Fig. 1e; Extended Data Table 3, which contains the full list of the 1300 proteins), including a PM enriched for platelet and lymphocyte activation (PM6; Extended Data Table 5), as well as other PMs enriched for tissue-specific proteins as annotated in the Human Protein Atlas^16^, such as bone marrow proteins in PM3 (OR = 23.70, adj. *p* = 1.7x10^−6^) and spleen proteins in PM2 (OR = 11.18, adj. *p* = 4.6x10^−5^) (Extended Data Table 6). In contrast to the highly modular nature of blood transcriptomic measurements (Fig. 1d), a large fraction (48%) of the proteins fell into the “gray” module, which contains “singleton” proteins that did not exhibit sufficient correlation with other proteins to be incorporated in a module (Fig. 1e). Interestingly, the gray module proteins were enriched for those expressed in the liver (OR = 4.67, adj. *p* = 9.68x10^−8^), small intestine (OR = 3.71, adj. *p* = 0.011), and adipose tissue (OR = 4.00, adj. *p* = 0.045) (Extended Data Table 6). These observations are consistent with the notion that whole blood transcriptomic data mainly capture variation in circulating immune cell frequencies and cellular states that give rise to correlated, modular gene expression structures, while circulating protein levels reflect more diverse sources of variation, including those from circulating blood cells but also from tissues and potentially their status such as inflammation. The blood transcriptomic and serum protein measurements thus provide orthogonal, complementary information and together enable comprehensive assessment of phenotypically diverse individuals.

**Extended Data Figure 2.**
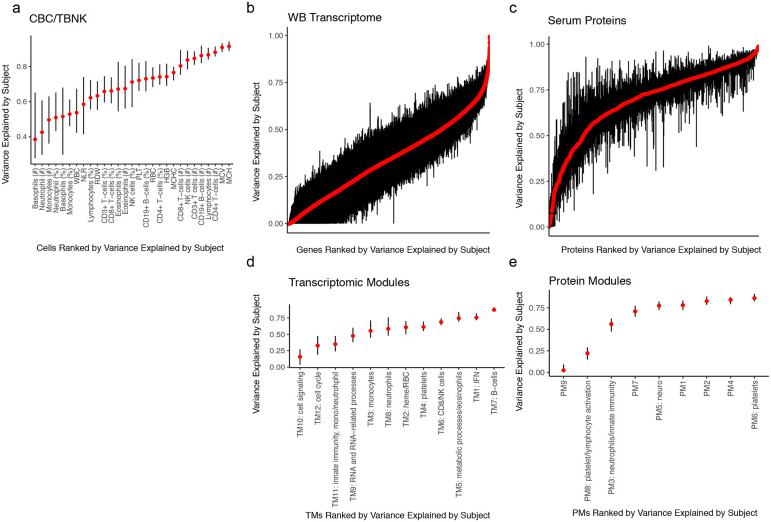
Jackknife resampling shows robustness of variation explained by subject covariate in mixed effect model A jackknife was performed subsampling 80% of subjects with repeat samples and 80% of subjects without repeat samples to assess robustness of intra-patient stability estimates for cell frequencies (**a**), gene expression (**b**), serum protein data (**c**), gene expression modules (**d**), serum protein modules (**e**). 100 replicates of subsampling were performed. Points represent mean variance explained by subject across all replicates and error bars denote 95% confidence intervals (2.5 % and 97.5 % quantiles across jackknife replicates). CBC = complete blood count. TBNK = lymphocyte (T, B, NK cell) phenotyping.

**Extended Data Figure 3.**
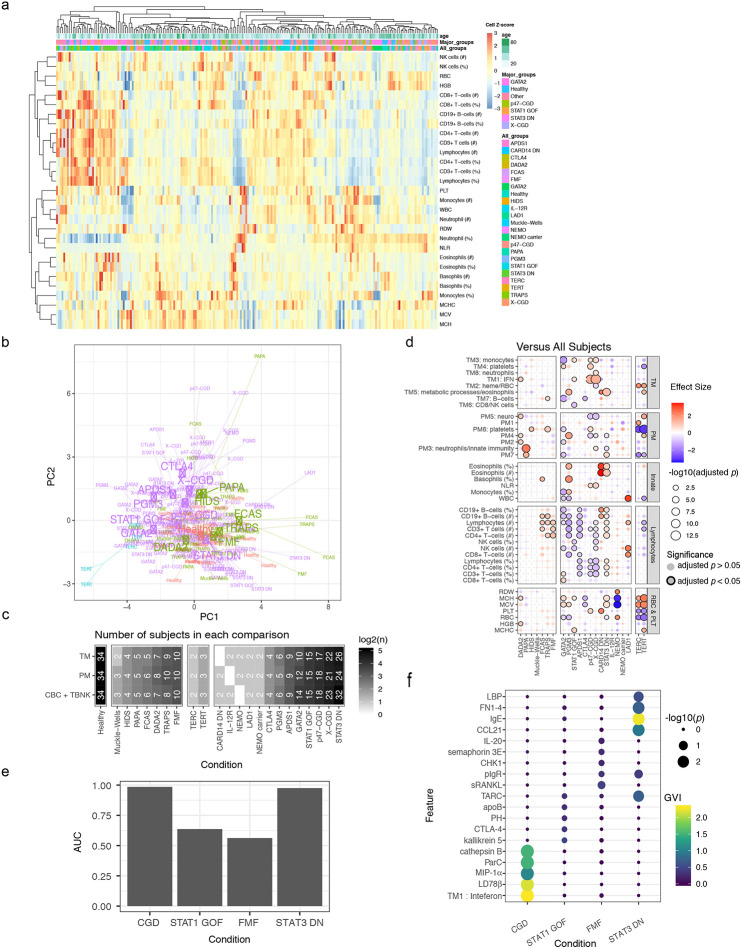
Supporting data for the disease-associated molecular and cellular signatures. **a,** Heatmap of complete blood count (CBC) and lymphocyte (T, B, NK cell) phenotyping (TBNK) parameters (rows) across patients and healthy subjects (columns); columns and rows are ordered by hierarchical clustering. Top annotation row shows the age of the subject, middle row shows the large condition groups (n > 10 subjects), and third row shows all condition groups regardless of number of subjects. **b,** Patients and healthy subjects shown in PC1 and PC2 space of CBC and TBNK parameters. Each parameter was standardized to unit variance and mean of zero prior to computation of the principal components. The text denotes the subject’s condition, and the color denotes larger condition groups. Large dots and text denote the centroid of that disease group. Only conditions with greater than three subjects have a centroid shown. AI = autoinflammatory diseases. Telo = telomere disorders. PID = primary immunodeficiencies. **c,** Table of sample sizes for each data modality-condition group combination. TM: whole blood transcriptomic modules; PM: protein modules. **d,** Similar to Fig. 2a but comparing each condition to all other conditions (healthy subjects are removed from the analysis). **e,** Barplot of Receiver Operating Characteristic Area Under the Curve (AUC) for conditions-versus-all-other-conditions Random Forest classifiers using all features as input. Classifiers were trained only for the four condition groups with the most subjects (healthy subjects were removed from the analysis); however, subjects from all other disease groups were used as the negative samples for each classifier. **f,** Plot of −log 10 adjusted *p* values and global variable importance (GVIs from the Random Forest models) of features in the classifiers for the four most represented disease groups. The plot is subset to the union of the top five predictive features for each condition.

Multiple sources contribute to variations in the level of a parameter (e.g., cell frequency or WGCNA module score), including those associated with disease and medications as well as inter-subject and temporal differences within individuals. Leveraging data from 63, 62, and 64 subjects for the cell frequencies, whole blood transcriptomics, and serum proteins, respectively, from whom we had collected more than one sample over time (spanning 5 days to roughly 1 year from 19 disease groups and healthy subjects, 25% quantile = 86 days, median = 130 days, 75% quantile = 181 days), we fit a variance partition model^17^ to estimate the relative contributions from the following sources: differences associated with disease, differences among patients with the same disease, medication/treatment effects, and intra-patient variations over time (Fig. 1f,g). A large fraction of the parameters, including blood transcripts and especially circulating proteins, was temporally stable within individual patients, i.e., the systematic differences between patients were larger than those in the same patient over time as indicated by the larger variance explained by the patient covariate (Fig. 1f,g; Extended Data Fig. 1e-g). Major medication categories, including steroids and immunosuppressants, could only account for a small fraction of the variance in most parameters (Extended Data Fig. 1h), suggesting that immune states of individuals were not broadly affected by these medications. Also unexpectedly, but consistent with the substantial temporally stable inter-subject variations, the differences between patients with the same disease (inter-subject variance explained by the patient) were often larger than the disease effects (i.e., group level average differences between disease and healthy: variance explained by the disease/condition label) for most of the serum protein and transcriptomic parameters (Fig. 1g). Jackknife analysis indicated that the variance explained by subject for all features is robust to sampling noise, particularly for the features with the highest variation explained by subject (Extended Data Fig. 2). Consistently, patients did not cluster by disease labels based on CBC/TBNK data alone, with healthy subjects intermixed with disease groups (Extended Data Fig. 3a,b), indicating that CBC and basic immune cell frequency data alone are insufficient to delineate health and disease. Together, these data suggest that factors such as the environment and exposure history play an important role in shaping the immune state of an individual, even in adult patients with highly penetrant monogenic conditions.

### Pan-disease and disease-specific signatures

**Figure 2.**
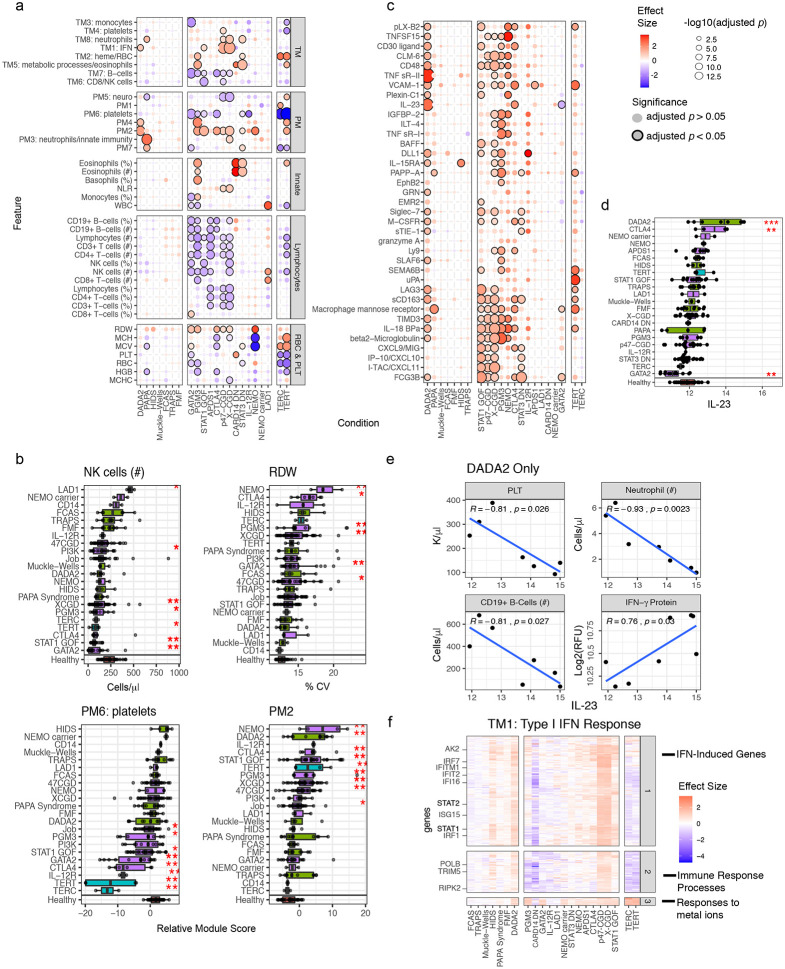
Molecular and cellular signatures of individual monogenic diseases. **a,** A bubble plot of temporally stable (>50% variance explained by subject) complete blood count (CBC) and lymphocyte (T, B, NK cell) phenotyping (TBNK) parameters, and serum protein and transcriptomic module scores (rows) vs. the disease groups (columns). Columns and rows are ordered by hierarchical clustering (columns/diseases were clustered within major groups, i.e. primary Immunodeficiencies, autoinflammatory diseases, and telomere disorders). The bubble color corresponds to the effect size (estimated difference between patients in the disease group vs. matching healthy subjects via a linear model) for each group while controlling for age, gender, and whether the patient was acutely ill during sampling. The size of the bubble reflects the adjusted *p* value associated with the fitted t-statistic and the presence of black outlines around the bubble denotes an adjusted *p* value < 0.05. Red boxes highlight specific parameters discussed in the text. TM = whole blood transcriptomic modules. PM = serum protein modules. IFN = interferon. NLR = neutrophil-to-lymphocyte ratio. WBC = white blood cell count. MCHC = mean corpuscular hemoglobin concentration. HGB = hemoglobin. RDW = red cell distribution width. PLT = platelet count. MCH = mean corpuscular hemoglobin. MCV = mean corpuscular volume. RBC = red blood cell count. NK = natural killer. **b**, Boxplots of NK cell count, RDW, and module scores of PM2, and PM6 (enriched for platelet-related factors) across all disease and healthy groups in the study. The healthy subject group is shown separately at the bottom. *P* values computed from linear models used in (**a**). *adjusted *p* value < 0.05, **adjusted *p* value < 0.01, ***adjusted *p* value < 0.001. Box plot center lines correspond to the median value; lower and upper hinges correspond to the first and third quartiles (the 25th and 75th percentiles), and lower and upper whiskers extend from the box to the smallest or largest value correspondingly, but no further than 1.5X inter-quantile range. **c,** Similar to (**a**) but limited to the PM2 member proteins (rows). The red box highlights IL-23, the distribution of which is shown in boxplot in (**d**). **d,** Similar to (**b**) but for IL-23 relative serum protein level (as measured by the Somalogic platform) across all disease conditions and healthy subjects in the study. **e,** Scatterplots showing the correlation between the relative serum protein level of IL-23 (as measured by the Somalogic platform) and the indicated peripheral blood cell frequencies/counts and the IFN-γ relative serum protein level (lower right plot) for DADA2 patients in the study. Pearson correlation coefficient and associated *p* value shown. **f,** Heatmap of effect sizes from linear models of individual transcripts (rows) from TM1 (enriched for interferon-stimulated genes) transcriptomic module. All transcripts in the module are shown without filtering based on significance. The cell color corresponds to the effect size (estimated log fold-change relative to healthy subjects) for each disease group (columns) while controlling for age, sex, and whether the patient was acutely ill during sampling. The genes are clustered into three groups as indicated on the right. Example gene names are highlighted on the left. IFN = interferon.

We next derived and compared disease signatures, although our aim was to generate new hypotheses rather than “deep diving” mechanistically into any specific monogenic disease. We used linear models to derive signatures of individual disease conditions in comparison to matching healthy subjects accounting for age, sex, and major medication groups (Fig. 2a; Extended Data Fig. 3c). Despite the diversity of conditions, we detected signatures shared across diseases. These shared signatures had consistent directions of change across multiple diseases, including increases in red cell distribution width (RDW; a measure of the variation of erythrocyte volume^18^), TM2 (enriched for heme biosynthesis), and PM2, as well as decreases in TM6 (enriched for NK cells and CD8+ T-cells), NK cell frequencies, and PM6 (enriched for platelet related factors) (Fig. 2a,b; Extended Data Tables 7-9). RDW is known to be associated with all-cause mortality and several common diseases, including cardiovascular disease and cancer^19^, but it has not been assessed simultaneously across multiple pathologies including the monogenic diseases analyzed here. Proteins in PM2 spanned several inflammatory pathways (Extended Data Table 3), including interleukin-23 (IL-23), tumor necrosis factor α soluble receptors 1 and 2, interferon (IFN)-related or -induced proteins [e.g., IP-10/CXCL10, I-TAC/CXCL11, monokine induced by gamma (MIG)/CXCL9], and the shed receptor sCD163 that might reflect macrophage activation in tissues^20^. Together, these signals may reflect both systemic and tissue inflammation shared across diseases.

As an example of how our comparative analysis may be explored to reveal disease-specific insights, we identified signatures more specific to individual or subgroups of diseases. For example, the PM2 score was highly elevated in deficiency of adenosine deaminase 2 (DADA2) patients and several PIDs such as STAT1 gain-of-function (STAT1 GOF) and X-linked chronic granulomatous disease (X-CGD), relative to healthy subjects (Fig. 2a,b; Extended Data Table 8). IL-23, a member of PM2, was elevated in DADA2 (Fig. 2c,d; Extended Data Table 10), even though it is not a known marker of this disease. IL-23 was positively correlated with IFN-γ in DADA2 patients (Fig. 2e), consistent with the fact that IL-23 can induce IFN-γ production in several cell types such as γδ and CD8+ T-cells^21^. Although we verified that this increase in IL-23 was not driven purely by changes in cell frequencies by fitting an additional model controlling for major cell subset frequencies (Extended Data Table 12, see Methods), DADA2 patients with high IL-23 tended to have decreased platelets, neutrophils, and total B-cells (Fig. 2e). These phenotypes are consistent with bone marrow biopsies from some of these DADA2 patients that showed decreased cellularity and B-cell precursors. Interestingly, like DADA2, some GATA2 deficiency (GATA2) patients also had lower peripheral blood cell counts but *decreased* levels of circulating IL-23 (Fig. 2c), suggesting that the connection between circulating IL-23 level and bone marrow status in DADA2 patients is distinct from that in other diseases with bone marrow failure or low peripheral cell count phenotypes.

Elevated type I IFN (IFN-I) blood transcriptional signatures have been found in monogenic and polygenic inflammatory diseases such as Aicardi-Goutières syndrome and SLE, respectively^22,23^. Here DADA2, STAT1 GOF, X-CGD, and p47^phox^CGD (p47-CGD) had clear IFN-I signatures as reflected by elevation in TM1 (FDR < 0.2; Fig. 2a, Extended Data Table 9). This is to be expected for the STAT1 GOF patients given their elevated STATl-dependent signaling^24^. However, the CGDs, not typically known as interferonopathies^22^, had the most elevated TM1 scores compared to healthy (Extended Data Table 9), which were also significantly higher than STAT1 GOF (X-CGD vs STAT1 GOF: logFC = 0.83, *p* = 0.001; p47-CGD vs STAT1 GOF: logFC = 0.82, *p* = 0.002). Relative serum concentrations of the IFN-inducible protein I-TAC/CXCL11, as well as STAT1 itself, were higher in X-CGD and STAT1 GOF patients relative to healthy subjects (Extended Data Table 10), with circulating STAT1 protein concentrations significantly higher in X-CGD compared to STAT1 GOF (logFC = 0.83, *p* = 0.006). Consistently, IFN-inducible transcripts in TM1 tended to be elevated in both the CGDs and STAT1 GOF patients compared to healthy, but again the elevations appeared stronger in the CGDs than the STAT1 GOF (Fig. 2f, Extended Data Table 11). We additionally verified that this increase in TM2 score was not driven purely by changes in cell frequencies by fitting an additional model controlling for major cell frequencies (Extended Data Table 12, see Methods). Together, these results suggest that IFN-I signatures and related pathways may be a good source of biomarkers and therapeutic targets for CGD.

In addition to examining differences in relation to healthy subjects, we also compared each disease against all other diseases excluding the healthy subjects. Surprisingly, this other-disease-as-background map was qualitatively similar to the healthy-as-background map (Extended Data Fig. 3d). For example, the autoinflammatory diseases tumor necrosis factor receptor-associated periodic syndrome (TRAPS), familial cold autoinflammatory syndrome (FCAS; NLRP3-associated autoinflammatory disease-mild) and familial Mediterranean fever (FMF) as a group differed from the healthy subjects and other diseases by similar signatures, including lymphocyte and B-cell counts that trended higher than other diseases, which to the best of our knowledge has not been described for this group of diseases. These disease-specific signatures suggest that predictive models could also be built to help identify possible diagnoses for patients. Indeed, Random Forest (RF) classifiers built for the major disease groups (Extended Data Fig. 3e,f) revealed that STAT3 dominant-negative (STAT3 DN) disease patients (also known as autosomal dominant hyper-IgE syndrome or Job’s Syndrome) could easily be differentiated from other patients in the cohort based on cross-validation analysis (0.98 AUC, STAT3 DN n = 21, Other n = 127), as could the p47-CGD/X-CGD patients (0.99 AUC, CGD n = 37, Other n = 111). In contrast, predictive performance was poorer for STAT1 GOF (0.64 AUC, STAT1 GOF n = 15, Other n = 133) and FMF (0.56 AUC, FMF n = 10, Other n = 138), which may reflect disease and patient heterogeneity, some of which might not be well captured by the parameters measured, or because FMF patients may have been sampled largely at clinically quiescent time points^25^. Together, our data provide a rich resource for the biomedical community and highlight shared and disease-specific cellular, transcriptional, and serum protein signatures of diverse monogenic immune-mediated diseases. The shared signatures in particular point to commonly dysregulated pathways and processes in the immune system independent of disease-specific pathologies.

### Integration of transcriptomic and serum protein personal immune profiles revealed an emergent axis of immune health

**Figure 3.**
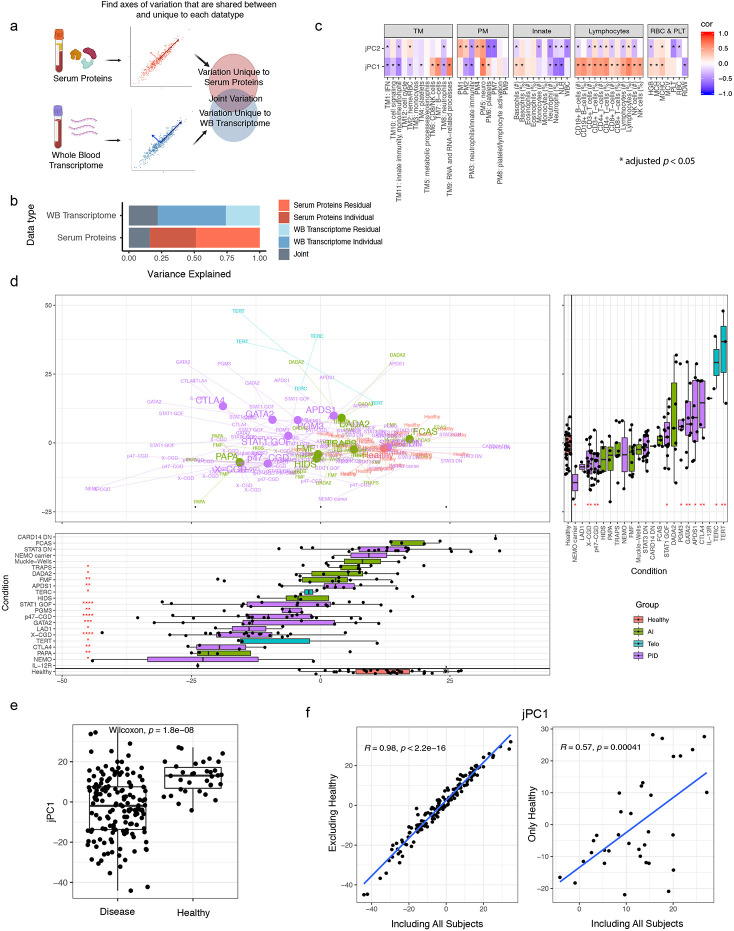
Bottom-up integration of transcriptomic and serum protein personal immune profiles reveals an emergent axis of immune health. **a,** Conceptual overview of JIVE analysis integrating whole blood transcriptome and serum protein data. JIVE was performed using the subject-level data (n=188 subjects who had both serum protein and whole blood transcriptomic data). **b,** Variation explained by the joint (grey – shared by both data types), individual data type (darker blue and red for transcriptome and protein data, respectively), and residual latent factors (lighter blue and red for transcriptome and protein data, respectively) in JIVE analysis. **c,** Heatmaps showing Pearson correlation between jPCs (rows) and major peripheral immune parameters and module scores (columns). Red denotes positive correlation and blue denotes negative correlation (*adjusted *p* value < 0.05, FDR adjustment performed across all comparisons together). Correlation was computed using the subject-level data (n = 182 subjects who had serum protein, whole blood transcriptomic, and CBC/TBNK data). TM = whole blood transcriptomic modules. PM = serum protein modules. IFN = interferon. NLR = neutrophil-to-lymphocyte ratio. WBC = white blood cell count. MCHC = mean corpuscular hemoglobin concentration. HGB = hemoglobin. RDW = red cell distribution width. PLT = platelet count. MCH = mean corpuscular hemoglobin. MCV = mean corpuscular volume. RBC = red blood cell count. NK = natural killer. **d,** Projection of patients and healthy subjects onto the jPC1 vs. jPC2 space. N = 154 and 34 disease and healthy subjects, respectively. Text label shows the disease group to which the patient belongs. Colors denote disease categories involving larger groups of conditions. Large dots and text denote the centroid (mean jPC1 and jPC2 values) of the indicated disease group. Only conditions with greater than three subjects have a centroid shown. Boxplots show projections onto single PC dimensions with patients grouped by disease condition (jPC1 below the centroid plot; jPC2 to the right of the centroid plot). Each subject’s score is represented as a single point. The healthy subject group is shown in red. (* *p* < 0.05, ** *p* < 0.01, *** *p* < 0.001, *p* values from two-sided Wilcoxon test). Box plot center lines correspond to the median value; lower and upper hinges correspond to the first and third quartiles (the 25th and 75th percentiles), and lower and upper whiskers extend from the box to the smallest or largest value correspondingly, but no further than 1.5X inter-quantile range. The healthy subject group is shown in red. (*p < 0.05, **p < 0.01, ***p < 0.001, *p* values from two-sided Wilcoxon test). AI = autoinflammatory diseases. Telo = telomere disorders. PID = primary immunodeficiencies. **e,** Boxplot of jPC1 scores comparing patients (all disease conditions combined) with healthy subjects [*p* value computed using two-sided Wilcoxon test; same set of subjects in panel (**d**)]. Box plot center lines correspond to the median value; lower and upper hinges correspond to the first and third quartiles (the 25th and 75th percentiles), and lower and upper whiskers extend from the box to the smallest or largest value correspondingly, but no further than 1.5X inter-quantile range. **f,** Scatterplot of JIVE PCs derived using all subjects vs. JIVE PCs derived using patients only by removing healthy subjects (left) or only healthy subjects alone (right). Spearman correlation and associated *p* value shown [n = 154 and 34 patients and healthy subjects, respectively; same as in panels (**d**) and (**e**)].

Our disease signature analyses suggest that both overlapping and unique information is provided by blood transcriptomic and circulating serum protein data. To assess whether the shared information between them can provide more integrated measures to examine individual patient-to-patient heterogeneity without knowledge of disease labels (Fig. 1b), we used JIVE^26^ to infer latent components shared among the temporally stable transcriptomic and serum protein parameters (Fig. 3a, see Methods). JIVE decomposes the data into components, including the shared information between both data types reported as “joint principal components” (jPCs) and information captured uniquely by each data type (individual principal components; iPCs).

**Extended Data Figure 4.**
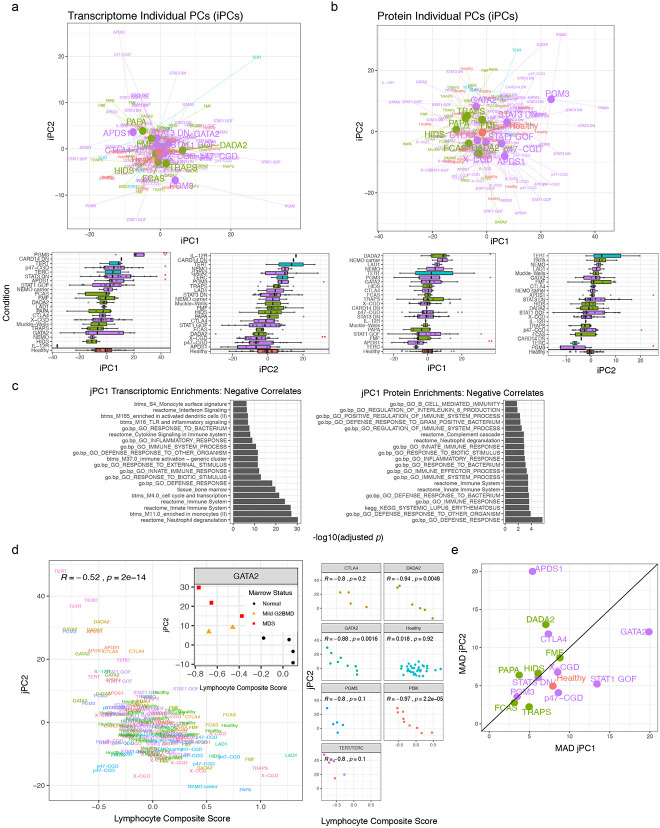
Characteristics of the individual and joint PCs from the JIVE analysis. **a,** Top panel: patients and healthy subjects shown in transcriptomic individual PC (iPC) 1 vs. iPC2 space. Large dots and text denote the centroid of that disease group. Only conditions with greater than three subjects have a centroid shown. Bottom panels: boxplots of individual transcriptomic iPC1 and iPC2. The rows correspond to the conditions and the color denotes larger condition groups. Box plot center lines correspond to the median value; lower and upper hinges correspond to the first and third quartiles (the 25th and 75th percentiles), and lower and upper whiskers extend from the box to the smallest or largest value correspondingly, but no further than 1.5X inter-quantile range. AI = autoinflammatory diseases. Telo = telomere disorders. PID = primary immunodeficiencies. **b,** Similar to (**a**) but showing the serum protein iPCs. **c,** Gene set enrichment of transcriptomic (left) and serum protein (right) features negatively correlated with jPC1 (enrichment calculated using CameraPR; genes/proteins ranked by the Spearman correlation with the JIVE PCs). Gene sets from KEGG pathways, GO biological process gene sets, Reactome pathways, and the blood transcriptomic modules and Human Protein Atlas tissue gene sets. **d,** Scatterplot of a hematopoietic composite score (see Methods) vs. jPC2. Left panel displays the trend across all patients including healthy subjects and the right set of panels focus on individual disease groups whose clinical presentation may include marrow failure or lymphopenia. Inset focuses on GATA2 patients, highlighting those with abnormal bone marrow biopsies. Spearman correlation and associated *p* values are shown. G2BMD = GATA2 deficiency-associated bone marrow disorder. MDS = myelodysplastic syndrome. **e,** Scatterplot of Median Absolute Deviation (MAD) of jPC1 and jPC2 scores for each condition in the study. A higher MAD corresponds to greater variation within a disease for that jPC.

JIVE revealed that approximately 20% of the variation (or information) in each data type was shared (Fig. 3b) with jPCs 1, 2 and 3 capturing 56%, 28% and 16% of the joint variation, respectively. The unique information in each data type could be further decomposed into 25 and 18 iPCs for the transcriptomic and serum protein data, respectively (Extended Data Fig. 4a,b; Extended Data Table 13). The top two transcriptomic data-specific iPCs reflected diverse processes and cell types, such as enrichments of neutrophil degranulation, monocytes, and IFN-I signatures. The top two protein-specific iPCs similarly exhibited enrichments for several functions, including extracellular matrix proteins, neurological processes and certain signaling pathway components (Extended Data Tables 14 and 15). These JIVE results suggest that not only can blood transcriptomic and serum protein data mutually reinforce each other based on the shared information present in jPCs (see below), each on its own can provide potentially non-redundant information and should thus be collected and analyzed together in human immune profiling studies.

We next focused on the shared jPC components because they captured information from both data modalities and thus provide robust information regarding personal immune states and patient-to-patient heterogeneity. jPC1 appeared to quantify the extent of *attenuation* in inflammation-related processes as evident by: 1) jPC1 was negatively correlated with the neutrophil-to-lymphocyte ratio, which is a known marker of systemic inflammation and elevated in acute infections and cancer^27,28^, and positively correlated with B- and T-cell frequencies (Fig. 3c; Extended Data Table 16); and 2) jPC1 was negatively associated with innate immunity, inflammation, and IFN related processes (Fig. 3c, Extended Data Fig. 4c, Extended Data Table 15). jPC2 was negatively associated with the counts of multiple cell lineages, including WBC, platelet, neutrophils, monocytes, lymphocytes, and hemoglobin (Fig. 3c, Extended Data Table 16), suggesting that it captured hematopoietic output capacity. Indeed, it was also negatively associated with a combined score derived from the above immune cell populations (Extended Data Fig. 4d). This negative association was especially apparent within the DADA2, GATA2, and activated PI3K delta syndrome 1 (p110δ; APDS1) patient groups (Extended Data Fig. 4d), consistent with the loss of one or more cell lineages being a shared characteristic of these diseases^29-32^. Interestingly, for GATA2, patients with the highest jPC2 scores were also more likely to have dysplastic marrow (Extended Data Fig. 4d), a known complication of the disease^30^.

We next placed individual patients onto the two-dimensional jPC1 vs. jPC2 space to visually examine inter-patient and inter-disease heterogeneity (Fig. 3d). Most disease groups and healthy subjects displayed narrower or comparable within-group variations along jPC2 than jPC1, but a few (DADA2, APDS1, CTLA4 haploinsufficiency) appeared to have higher jPC2 differences among patients (Extended Data Fig. 4e), which, at least for DADA2 and APDS1, is expected given that jPC2 reflects hematopoietic output and bone marrow pathologies are known to be variable in both groups of patients^33,34^. Consistent with the notion that jPC1 might reflect systemic inflammatory burden (or immune “health”) and the expectation that patients would have elevated inflammation and potentially poorer immune health, jPC1 score is significantly higher in healthy subjects than patients (Fig. 3e), and this was robust to adjusting for major cell frequencies (Extended Data Table 12). Intriguingly, however, healthy subjects alone spanned a wide range along jPC1, similar to or even exceeding that of patients within individual disease groups, suggesting that jPC1 might provide quantitative information on systemic inflammation among even clinically healthy individuals.

To test whether jPC1 emerged solely because of differences between sick patients and healthy subjects, we removed healthy subjects from our cohort and repeated the JIVE analysis. Strikingly, the resultant jPCs were highly correlated with those previously computed with HCs included (Fig. 3f; r = 0.98, 0.97, 0.92, respectively, for jPCs 1, 2, and 3). In fact, even if only healthy subjects were used to derive the jPCs, the resultant jPC1 was still significantly correlated with the original jPC1 derived from patients and HCs together (Fig. 3f). These results together suggest that the major emergent axis of immune variation within healthy subjects alone (i.e., derived in a totally unsupervised manner) is surprisingly similar to that obtained from sick patients with diverse monogenic immune-mediated diseases. These observations provide further support that this axis captures important information about immune health in diverse individuals.

In addition to the healthy subjects, most disease groups such as STAT3 DN, GATA2, and STAT1 GOF, spanned a wide range along jPC1 (Fig. 3d). The extensive overlap of healthy subjects and STAT3 DN patients is notable given that these patients could be easily distinguished from healthy subjects based on a few parameters as described in the disease classification analysis above (Extended Data Fig. 3e,f), suggesting that jPC1 captures immune health related phenotypes distinct from disease-specific deviations from healthy. On the “less healthy”, lower end of the jPC1 spectrum were CGDs; they also had extensive heterogeneity along jPC1, which is consistent with their wide spectrum of clinical presentations, including frequent infections, colitis, and pulmonary disease^35^, although further assessment would be needed to ascertain potential correlations between jPC1 and clinical phenotypes in larger patient cohorts. Patients with p47-CGD also trended higher than X-CGDs (*p* = 0.09, Wilcoxon test), consistent with the tendency for less severe disease in p47-CGD compared to X-CGD patients^36^. Together, our unbiased integration of blood transcriptomic and circulating protein data revealed an emergent axis of immune health that delineates both inter-disease and inter-subject heterogeneity in patient and healthy populations.

### A quantitative metric of human immune health

**Extended Data Figure 5.**
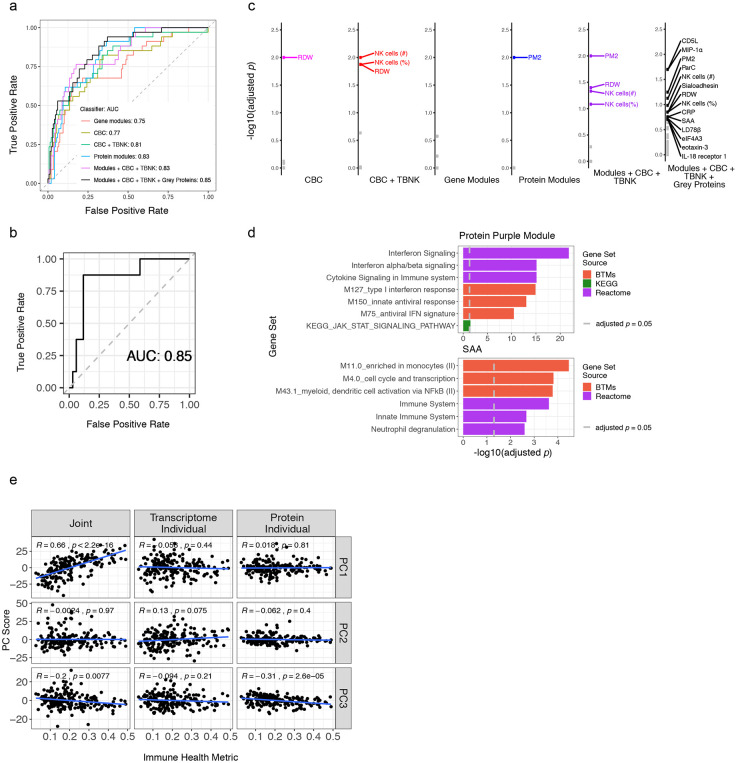
Supporting data for the development and characterization of the Immune Health Metric (IHM). **a,** Receiver Operating Characteristic (ROC) curves for Random Forest classifiers from LOOCV (leave-one-out-cross-validation) using temporally stable features of individual or the indicated combinations of data modalities. CBC = complete blood count. TBNK = lymphocyte (T, B, NK cell) phenotyping. **b,** ROC curve for the Random Forest classifier (the one trained on all data modalities in the primary dataset) applied to the set of unseen, independent set-aside patients and healthy subjects. **c,** Negative log10 adjusted *p* values (FDR) of Global Variable Importance of features in each Random Forest classifier. *P* values were determined through permutation (see Methods). Labels are shown for parameters passing an FDR cutoff of 0.2 for each classifier. FDR adjustment was performed on *p* values for parameters within a classifier. Features used in classifier are shown on x-axis. NK = natural killer. RDW = red cell distribution width. **d,** Enrichment of transcriptional surrogate signatures for the predictive features identified by the Random Forest classifier in Fig. 4b; gene sets from KEGG pathways, GO biological processes, Reactome pathways, and the blood transcriptomic modules (BTMs) were included for the enrichment analysis. SAA = serum amyloid A. **e,** Scatterplots with regression lines and associated Pearson correlations and *p* values of subjects’ Immune Health Metric (IHM) scores vs. the first 3 PC scores from the jPCs, transcriptomic individual PCs (transcriptomic iPCs), and serum protein individual PCs (proteomic iPCs). N = 182 subjects with both jPC and IHM scores. Pearson correlation and associated *p* value are shown.

**Figure 4.**
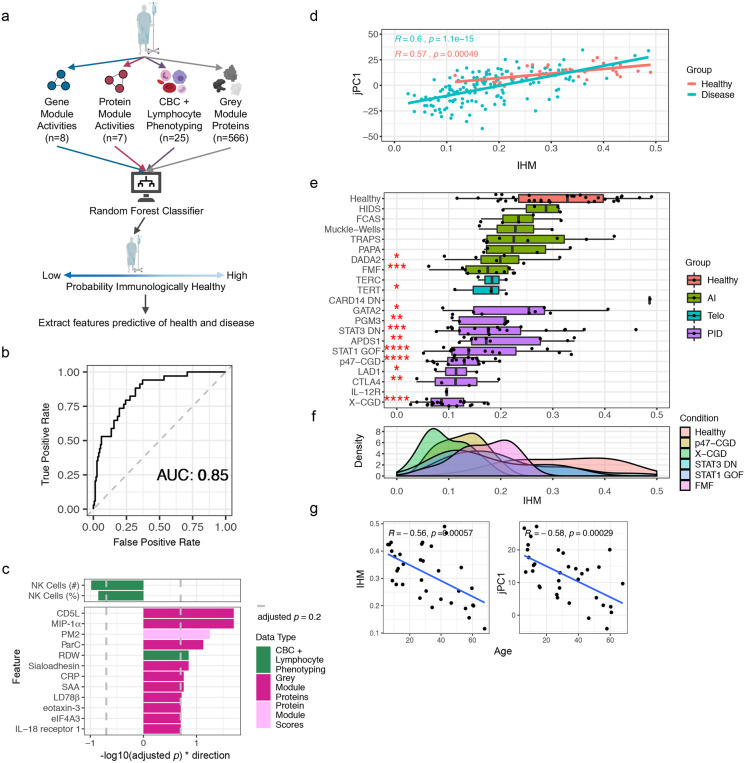
Top-down supervised machine learning classification analysis independently reveals an immune health metric highly concordant with that from unsupervised analysis. **a,** Conceptual overview of the supervised machine learning analysis of healthy vs. disease patients using Random Forest classifiers to obtain a probability score of immunological health [the Immune Health Metric (IHM)]. The number of temporally stable features used from each data modality is shown. Models were trained using the subject-level data (n = 182 subjects with serum protein, whole blood transcriptomic, and CBC/TBNK data). **b,** Receiver Operating Characteristic (ROC) curve for distinguishing healthy subjects vs. patients using the approach shown in (**a**). **c,** Barplot of the −log10 adjusted *p* values for features passing a 0.2 FDR significance cutoff (grey dashed line; *p* values estimated through permutation testing of Global Variable Importance from the Random Forest classifiers); these are top features contributed to the classifier used to derive the IHM. Direction was determined as the sign of the average difference between heathy subjects and patients from all disease groups. **d,** Scatterplot showing correlation between IHM score and the jPC1 scores across subjects. Least squares regression lines included for healthy subjects with correlation statistics shown. 95% confidence interval of the estimated conditional mean is shown. N = 148 and 34 disease patients and healthy subjects, respectively. **e,** Boxplots of IHM scores of individual subjects grouped by condition (disease and healthy groups). The healthy group (top row) is shown in red; the statistical significance of the comparison between the condition and the healthy groups is shown for conditions that tested significant (*p < 0.05, **p < 0.01, ***p < 0.001, *p* values from two-sided Wilcoxon test). Box plot center lines correspond to the median value; lower and upper hinges correspond to the first and third quartiles (the 25th and 75th percentiles), and lower and upper whiskers extend from the box to the smallest or largest value correspondingly, but no further than 1.5X inter-quantile range. AI = autoinflammatory diseases. Telo = telomere disorders. PID = primary immunodeficiencies. **f,** Similar to (**e**), but here showing smoothed density of IHM scores for each of the groups with at least 10 subjects. **g,** Scatterplots with trendlines showing the age dependence of the IHM and jPC1 in healthy individuals only (Spearman correlation and *p* values shown; n = 34 healthy subjects with serum protein, whole blood transcriptomic, and CBC/TBNK data).

The emergence of pan-disease signatures (Fig. 2a) and an immune health axis, jPC1, (Fig. 3d) prompted us to assess whether supervised machine learning could help refine our immune health metric and the associated correlates of health and disease. We tested several RF healthy-versus-all-disease classifiers using temporally stable parameters as inputs, each using a different combination of data modalities (Fig. 4a) and assessed its performance with leave one out cross-validation (LOOCV). The classifier using all data modalities [including the use of singleton, grey module proteins (Fig. 1e)] had the best performance (Fig. 4b, Extended Data Fig. 5a). It showed similar prediction performance in the independent (thus never-been-seen) set of patients and healthy subjects we set aside immediately after data generation but before any analysis began (these subjects were not included in the initial LOOCV evaluation or any of the analyses described in this manuscript except here in this independent robustness check; Extended Data Fig. 5b). This classifier revealed top parameters that contributed to the prediction [as measured by permutation tests of the global variable importance (GVI) – Extended Data Table 17]. These include RDW and parameters capturing systemic inflammation (sialoadhesin, C-reactive protein, PM2) and myeloid cell/macrophage signals (MIP-1α, LD78β), as well as the frequency of circulating NK cells (Fig. 4c, Extended Data Fig. 5c,d). These together revealed common deviations of disease from normal and are broadly concordant with the qualitative pan-disease signatures above (Fig. 2a).

In essence, our RF classifier had learned from a diverse set of monogenic diseases (i.e., as “negative” examples of health) against healthy subjects (“positive” examples) what a healthy immune system should (or should not) look like. Thus, we next used our classifier to assign each sample an “immune health metric” (IHM) score that reflects the probability that the sample belongs to the healthy group (see Methods, Extended Data Table 18). Despite jPC1 being derived in an unsupervised manner (i.e., without labeling the subjects with their disease/condition or healthy status), the IHM was highly correlated with jPC1 in patients with disease alone or in the healthy subjects only (Fig. 4d), but less so with the other jPCs (Extended Data Fig. 5e). As seen with jPC1 (Fig. 3d,e), the healthy subjects displayed a broad range of IHM scores (ranging from the very healthy to presumably the less healthy), but their median IHM score was significantly higher than that of most disease groups (Fig. 4e,f). Furthermore, consistent with the intuitive notion that immune health declines with age given that older individuals have elevated risk of immune-mediated diseases and tend to respond more poorly to infections and vaccinations compared to the young^37^, the IHM score and jPC1 were both negatively correlated with age in healthy individuals (Fig. 4g). Since certain cell frequencies are known to decline with age^37^, we verified that the IHM was correlated with age in healthy individuals even after controlling for cell-frequencies (Extended Data Table 12). Additionally, the IHM classifier could not have directly learned age-associated signals by training on patients versus healthy subjects because these two groups had indistinguishable age distributions in our cohort (KS test, D = 0.17, *p* = 0.41, Extended Data Fig. 1a,b). This negative age association also suggests that older healthy subjects resembled sick patients according to the IHM and age is a major contributor to IHM variability in the clinically healthy population. Thus, supervised (resulted in the IHM) and unsupervised (resulted in jPC1) analyses converged to a concordant metric of immune health.

### IHM is associated with common immune-mediated disease, vaccine responses in the elderly, and serum protein changes in healthy aging

**Extended Data Figure 6.**
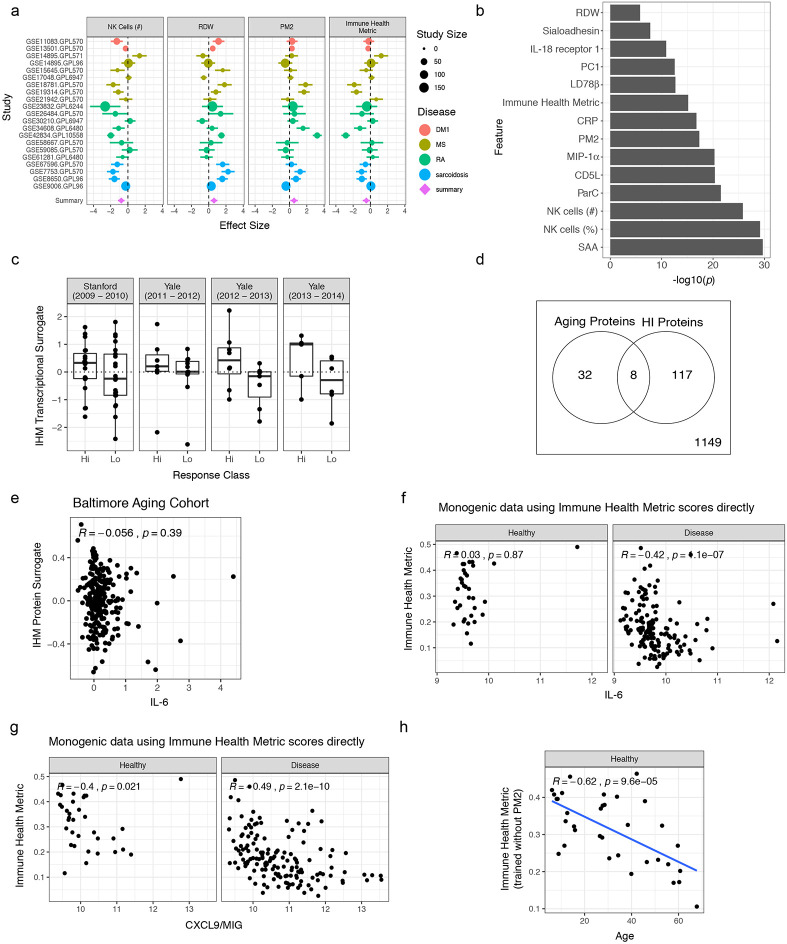
Supporting data for assessing the Immune Health Metric (IHM). **a,** Forest plot showing the effect sizes and associated standard errors in each study in the meta-analysis for a selection of the transcriptional surrogate signatures capturing the status of the indicated parameters (e.g., NK cell number). Summary meta-effect sizes shown at the bottom. Size of circles indicates the relative sample numbers of each study. Effect sizes correspond to average differences between disease and healthy, thus a positive effect size indicates that the parameter was elevated in disease compared to healthy on average. Error bars show the 95% confidence interval (1.96 * standard error) in the meta-analysis. **b,** Barplot of −log10 *p* value (two-sided Wilcoxon rank sum test) to assess whether genes in a given transcriptional surrogate signature had significantly lower *p* values in the meta-analysis results compared with genes not in the signature. **c,** Boxplots showing the transcriptional IHM scores of high and low responders in individual studies from elderly vaccine meta-analysis. **d,** Venn Diagram showing the overlap between proteins in the IHM protein surrogate signature and the original aging signature reported in the Baltimore Aging Study (odds ratio and *p* value from the one-sided Fisher’s exact test used to test the significance of the overlap). **e,** Scatterplot displaying the relationship between the IHM protein surrogate score and serum IL-6 relative serum protein concentration (as measured by the Somalogic platform) in the Baltimore Aging study (Spearman correlation and associated *p* value shown; n = 240). **f,** Scatterplots showing the relative serum level of IL-6 (as measured by the Somalogic platform) and the IHM in healthy subjects (left) and patients (right) in this study (Spearman correlation and associated *p* values shown). n = 148 and 34 disease and healthy subjects, respectively. **g,** Scatterplots showing association between the relative serum level of CXCL9/monokine induced by gamma (MIG; as measured by the Somalogic platform) and the IHM in the healthy subjects (left) and patients only (right) in our study (with Spearman correlation and *p* value shown). n = 148 and 34 disease and healthy subjects, respectively. **h,** The IHM was re-derived but without including PM2 (which contains CXCL9/MIG and correlated proteins) during training or testing. Scatterplot shows the correlation between age and this alternative IHM (without PM2) in the healthy subjects only (with Spearman correlation and *p* value shown; n = 34).

**Figure 5.**
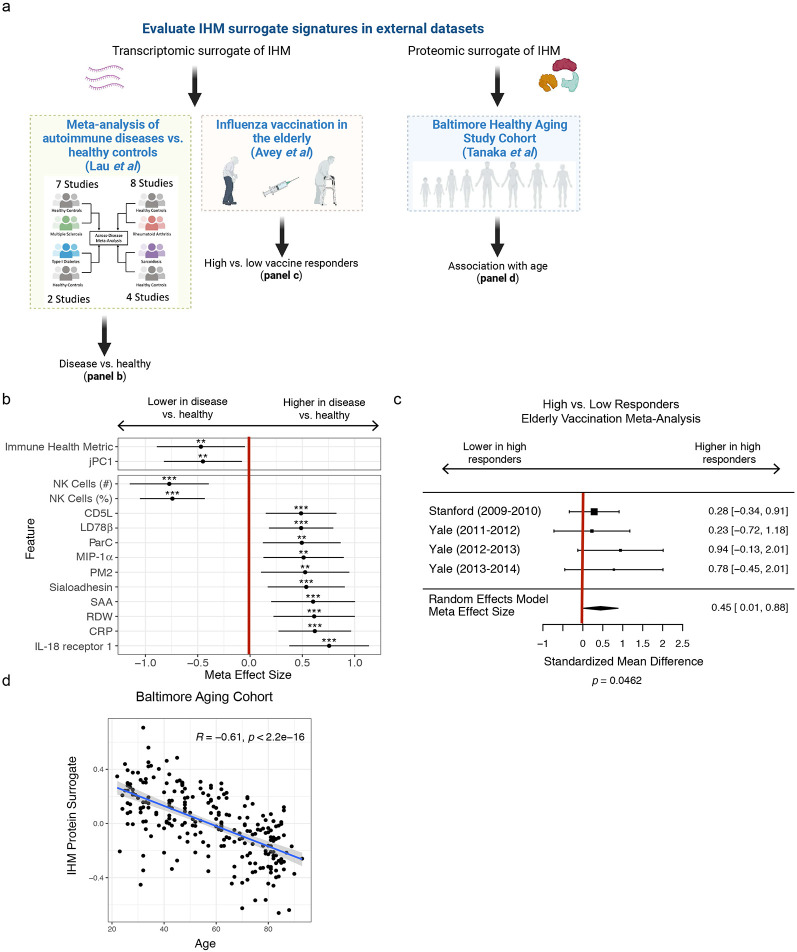
Assessing the IHM in independent datasets **a,** Graphical depiction of the creation of blood transcriptional and protein surrogate signatures followed by (from left to right): 1) meta-analysis of four common, non-monogenic autoimmune/inflammatory diseases across 21 independent studies, 2) meta-analysis comparing high vs. low responders in influenza vaccination in the elderly, and 3) validation of the IHM and healthy aging association using an independent cohort. **b,** Plot of meta effect sizes (average difference between disease and healthy groups) for each surrogate gene signature tested using the meta-analysis, including the IHM itself with a statistically significant negative effect size (i.e., it is lower in disease than healthy). The point shows the estimated effect across all studies used in the meta-analysis and error bars show the 95% confidence interval (1.96 * standard error) in the meta-analysis. **c,** Forest plot of effect sizes from the meta-analysis across four independent influenza vaccination cohorts of elderly subjects testing whether the IHM transcriptional surrogate signature evaluated at baseline before vaccination was associated with antibody titer responses to seasonal influenza vaccination in elderly individuals (i.e., whether those with better immune health according to the IHM had higher antibody responses.) Effect sizes in each study (squares), their 95% confidence interval (1.96 * standard error, error bars around square), the overall meta effect size (diamond) combining evidence across the four cohorts and the standard error of the meta-effect (width of diamond) are shown. Size of square denotes the relative number of subjects in that study. **d,** Scatterplot with trendline showing the negative correlation between chronological age and the circulating protein-based IHM surrogate signature scores (see Methods – the circulating protein IHM surrogate was developed using data from our cohorts only) in healthy subjects from the independent Baltimore Aging Study (Tanaka *et al.*, 2018). N = 240 subjects.

To assess the generalizability of the IHM beyond the monogenic diseases we studied, we sought to validate and further characterize the biological relevance of the IHM using independent datasets (Fig. 5a). First, we assessed the IHM in common autoimmune/inflammatory diseases distinct from the rare monogenic ones we examined above by using blood transcriptomic data from a published meta-analysis of 21 independent human datasets of type 1 diabetes, sarcoidosis, RA, and multiple sclerosis (Extended Data Table 19)^38-40^. We estimated the coherent deviation (meta-effect size) between disease and healthy subjects across the four diseases for every transcript and the transcriptional signature scores of the IHM, jPC1, and the top predictive markers from the IHM (the IHM and jPC1 signatures comprise blood transcripts correlated with the IHM or jPC1 – herein referred to as the “IHM and jPC1 blood transcriptional signatures”; Extended Data Table 20; see Methods). We found that these transcriptional signature scores were both significantly different between the four common diseases and healthy controls in the expected directions (Fig. 5b; Extended Data Fig. 6a,b; Extended Data Tables 21 and 22). Thus, the IHM can delineate health vs. disease in a different set of diseases common in the human population.

We next evaluated whether pre-vaccination immune health as reflected by the IHM might be predictive of responses to vaccination, a well-defined immune perturbation, and a potential *“in vivo”* readout of the consequences of having different levels of the IHM (Fig. 5a). We focused on the elderly population only because the extensive immune variability among the elderly is less well understood and baseline predictors of responses have been elusive in this population despite the fact that older individuals are known to have attenuated vaccination responses compared to the young^41^. Using meta-analysis of publicly available pre-vaccination blood transcriptomic data from four cohorts of older adults (61-96 years)^42^, we found that the IHM is indeed positively associated with antibody responses to influenza vaccination [summary effect size = 0.45 (weighted Hedge’s g between high and low responders across data sets), *p* = 0.046; Fig. 5c, Extended Data Fig. 6c]. Thus, the IHM could delineate baseline immune variation associated with vaccination outcomes among the elderly.

We next further assessed IHM-age associations in a published independent proteomic study (the “Baltimore Aging Study”) of 240 healthy subjects evenly distributed between the ages of 20 and 90^43^ (Fig. 5a). We derived circulating protein surrogates of the IHM (Extended Data Table 23) and found that the IHM protein surrogate score was indeed negatively correlated with age in this cohort (Fig. 5d). Interestingly, there was only a small overlap between the IHM circulating protein surrogates and those identified as associated with age in the original Baltimore study (Extended Data Fig. 6d), perhaps because the IHM is more reflective of aging-related immune health and inflammation^37^ while those identified in the original study captured aging signals from more biologically diverse sources. Furthermore, the IHM was not correlated with the level of circulating interleukin-6 (IL-6), a widely-studied cytokine linked to aging-related inflammation^44^, in healthy individuals from either the Baltimore Aging Study (Extended Data Fig. 6e) or our cohort (Extended Data Fig. 6f). However, IL-6 was correlated with the IHM when assessed in patients in our cohort (i.e., excluding healthy subjects; Extended Data Fig. 6f), partly because it was substantially elevated in some X-CGD and STAT1 GOF patients who had low IHM scores (data not shown). Thus, aspects of IL-6 related inflammation may be captured by the IHM in sick patients. In contrast, we did find that CXCL9/MIG, a marker known to be downstream of IFN-γ signaling and associated with aging-related inflammation^45^, is correlated with the IHM in both healthy subjects and patients alone (Extended Data Fig. 6g). However, the IHM remained negatively correlated with age independent of CXCL9/MIG (Extended Data Table 24) and its negative association with age did not change even when PM2, the protein module in the IHM that contained CXCL9/MIG (Fig. 2c), was removed during the derivation of the IHM (Extended Data Fig. 6h). Together, our results validate the utility and biological relevance of the IHM in distinct settings using independent datasets: a signature shared among common autoimmune and inflammatory diseases, a baseline correlate of vaccination responses in the elderly, and a biomarker of healthy aging.

### The cellular origin of the IHM transcriptional signature

**Figure 6.**
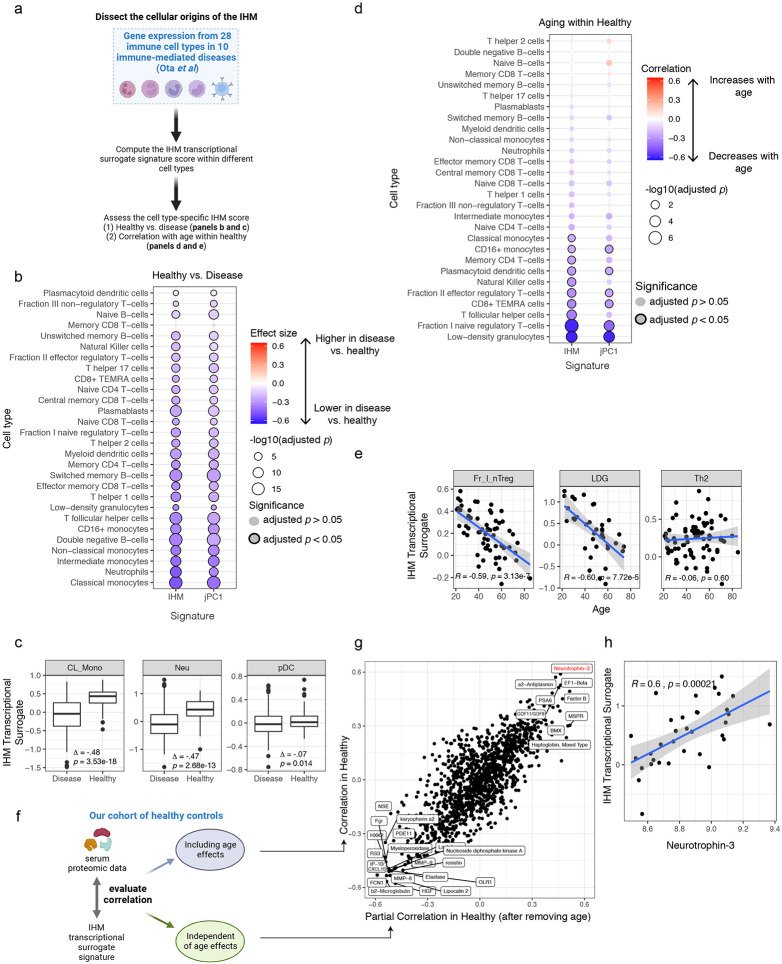
Cellular origin and circulating protein correlates of the IHM blood transcriptional surrogate signature **a,** Graphical overview of our analysis strategy for assessing 1) the differential expression of the IHM’s transcriptional surrogates between healthy and autoimmune disease, and 2) association with age, in each of 28 cell types from Ota *et al.* **b,** Bubble plot showing the effect sizes and statistical significance from the comparison of autoimmune diseases vs. healthy for the IHM and jPC1 transcriptional signature scores in 28 cell types from Ota *et al.* Effect sizes are denoted with the color scale shown. Significance is denoted by the size of the bubble and the presence of an outline. A negative effect size represents a decrease in the signature score in individuals with autoimmune disease relative to healthy. CD8+ TEMRA = CD8+ T effector memory CD45RA+ cells. **c,** Boxplots of IHM transcriptional surrogate signature scores comparing healthy controls vs. disease subjects from Ota *et al.* highlighting selected cell types from (**b**) CL_Mono: classical monocytes, Neu: neutrophil, pDC: plasmacytoid dendritic cells. Effect size (Δ) and *p* value are shown. **d,** Bubble plot showing Pearson correlation between age and the IHM (and jPC1) transcriptional signature scores in healthy individuals only, assessed separately for each one of the 28 cell types from Ota *et al.* Correlation strength is denoted by the color scale shown. Significance is denoted by the size of the bubble and the presence of an outline. A negative correlation represents a decrease in the signature score with older age. A higher signature score is associated with higher immune health. **e**, Scatterplots of IHM transcriptional surrogate signature scores vs. age in healthy controls from Ota *et al* highlighting selected cell types from (**d**) Fr_I_nTreg: Fraction I naive regulatory T–cells (Ota *et al*), LDG: low density granulocytes, Th2: T helper cells type 2. Pearson correlation and associated *p* value are shown. **f**, Graphical overview of the analyses behind the results shown in panel (**g**). We aim to identify circulating proteins that are correlated with the IHM whole blood transcriptional surrogate signature in our monogenic patients and assess whether the correlation (and thus the resulting protein correlates/surrogates) depends on age (thus without or with age effects removed). The age-dependent correlation is simply the correlation between the protein levels and the IHM transcriptional surrogate, whereas the age-independent refers to the partial correlation between these values after removing the effect of age with a linear regression model. **g**, Scatterplot showing the Spearman correlation values of serum proteins with the IHM transcriptional surrogate signature within healthy individuals only from the monogenic cohort. Raw Spearman correlations are shown on the y-axis, and partial correlations after removing the effect of age from the protein data and IHM transcriptional signature score are shown on the x-axis. The names of the 20 proteins with the highest absolute correlations on the x or y axes are shown. Neurotrophin-3 is highlighted in red. Correlations were computed with n = 34 healthy subjects only. **h**, Scatterplot of IHM transcriptional surrogate signature score vs. Neurotrophin-3 in healthy controls from this study (n=34). Spearman correlation and associated are *p* value shown.

To better understand the cellular origins of the IHM/jPC1 blood transcriptional signature, we utilized gene expression data of sorted peripheral immune cells from an independent study of 10 immune-mediated diseases (including RA and SLE) and healthy controls^5^. We computed the signature scores for the IHM and jPC1 within each cell type and tested whether these signatures were elevated in healthy controls compared to patients with immune-mediated diseases in the cohort (Fig. 6a; Extended Data Table 25). We found higher IHM and jPC1 signature scores in healthy individuals across nearly all the evaluated cell types (Fig. 6b,c), suggesting that the IHM and jPC1 reflect conserved transcriptional differences across a broad range of peripheral immune cells present in individuals with both polygenic and idiopathic immunological disease. These findings also further support the notion that the IHM/jPC1 and their constituent parameters are robust biomarkers of immune heath beyond rare monogenic immune diseases.

Since the IHM was associated with healthy aging (Fig. 4g, 5d), we also used only the healthy subjects from the gene expression data of sorted immune cells^5^ to assess what type of cells might have contributed to the age association. Compared to the disease-versus-healthy observations above, the IHM and jPC1 signature scores were negatively correlated with age in a subset of the cell types, most prominently in low density granulocytes (LDGs), a subset of naïve regulatory T-cells (Fr. I nTregs in Ota *et al*^5^), and certain T-cell subsets such as CD8+ effector memory T-cells expressing CD45RA (TEMRA) (Fig. 6d,e). These results suggest that while common blood transcriptional changes associated with immunological diseases are conserved broadly across multiple peripheral immune cell types (Fig. 6b; Extended data table 26), healthy aging-related decline in the IHM could be attributed to a more specific subset of these cell types. However, this observed difference could be partly driven by differences in statistical power given the larger effect and sample sizes in the disease-versus-healthy comparison. Taken together, the IHM blood transcriptional signature captures shared signals from multiple peripheral immune cell types and subsets.

### IHM captures immune variation in heathy individuals beyond age

**Extended Data Figure 7.**
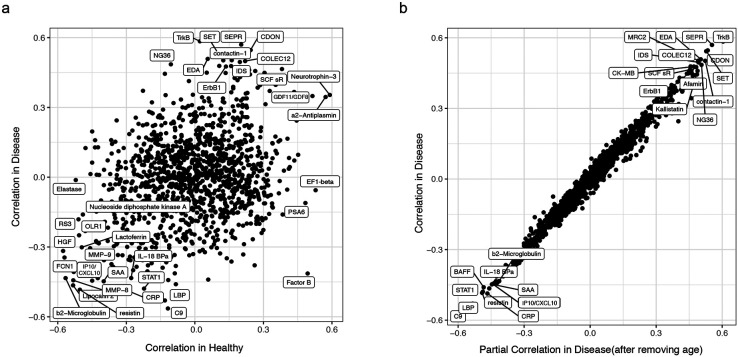
Supporting data for assessing the Immune Health Metric (IHM). **a,** Scatterplot showing the Spearman correlation of serum proteins with the IHM transcriptional surrogate signature within healthy individuals (x-axis) vs. disease individuals (y-axis) from the monogenic cohort. The names of the 20 proteins with the highest absolute correlations on the x or y axes are shown. Correlations were computed with n = 34 healthy and n = 154 for disease individuals. **b,** Similar to Fig. 6g but showing the correlation and partial correlation computed in subjects with disease only (n = 154).

Given the broad cell-type origin of the IHM, some of its serum protein surrogates/correlates (Extended Data Table 27) may represent cell extrinsic factors that could induce similar transcriptional profiles across different cell types – circulating serum proteins also represent easy-to-assay biomarker targets for routine clinical monitoring. Among the circulating protein correlates of the IHM, we noticed that some proteins were highly correlated with the IHM in both healthy subjects only and in patients (Extended Data Fig. 7a, Extended Data Table 27). These proteins include the IFN-induced IP-10/CXCL10 and beta-2 microglobulin, suggesting that interferons and related factors may be among the underlying cell-extrinsic inducers.

Given that age is a key contributor to IHM (and jPC1) variation, particularly in healthy subjects, and yet unexplained variation remains beyond age (Fig. 4g, 5d), we next assessed the extent by which the associations between serum proteins and the IHM depended on age (Fig. 6f). Surprisingly, they were largely independent of age (Fig. 6g). For example, certain proteins were highly correlated with the IHM, including IP-10/CXCL10 and other negative indicator of immune health (lower left-hand corner in Fig. 6g), regardless of age in healthy individuals (Fig. 6g, Extended Data Table 27) or in sick patients alone (Extended Data Fig. 7b, Extended Data Table 27). Interestingly, the positive correlates of the IHM (i.e., positive indicators of immune health – upper right-hand corner in Fig. 6g) were also independent of age. These include neurotrophin-3 (Fig. 6h) and GDF11/GDF8 (GDF11 is also known as BMP-11), both of which have critical developmental and potentially “rejuvenation” functions such as neurodevelopment, patterning, and angiogenesis^46-49^. Together, these observations suggest that factors beyond those linked to aging are shaping immune health (as reflected by the IHM) in clinically healthy individuals and the IHM variation among healthy subjects alone reflects both age-dependent and age-independent biology. Thus, learning from diverse rare diseases as “negative” examples of health also revealed a quantitative metric that captures meaningful variations in clinically healthy individuals.

## Discussion

Monogenic diseases are often studied in isolation due to their rarity, and thus the data and insight obtained from one condition cannot be easily compared to those of others. Here a unified approach was taken to simultaneously compare multiple rare immune-mediated conditions with natural genetic perturbations disrupting key pathways. To our surprise, despite penetrant genetic defects and clearly detectable common and disease-specific signatures, we observed that temporally stable, between-subject variation in cellular, transcriptomic, and circulating protein parameters dominates relative to the variation attributable to disease condition, medication, age, and sex. This observation is consistent with the clinical heterogeneity often observed even within single monogenic disorders^10^, suggesting that environmental, exposure history, and other genetic factors [e.g., genetic modifiers of primary causal mutations^50^] together play important roles in setting and maintaining personal immune states. Indeed, various immune parameters have been found to be temporally stable over months in healthy individuals; some of these inter-subject differences were associated with responses to perturbations such as vaccination and autoimmune disease flares^12-14^. Here we have extended these concepts and observations to diverse monogenic patients with high-penetrance deleterious mutations affecting immune functions.

In general, there were both shared and modality-specific information provided by the transcriptomic and circulating protein data, suggesting that both should be measured to capture personal biological states when possible. Importantly, our results using the protein and transcriptional signatures were largely independent of circulating immune cell frequency, which is a major driver of blood transcriptomic profiles. Some of the circulating protein modules we uncovered may also reflect tissue status, as was postulated previously in a large proteomic study of older individuals^51^. Our findings raise the possibility that a targeted set of parameters comprising select blood immune cell frequencies, proteins, and transcripts could be developed from a multi-disease cohort like ours with the goal of optimizing both information overlap (to increase robustness) and uniqueness (to capture diverse, informative biological states) to track the health and disease status of individuals in the general population.

Our dataset serves as a valuable resource for hypothesis generation and exploratory analyses by the research community. As an example, we revealed that IFN-stimulated gene transcripts were elevated in the blood of CGD patients and often at higher levels than in STAT1 GOF patients. This was unexpected given that STAT1 GOF patients are known to have increased STAT1 signaling and transcription of IFN-stimulated genes due to their gain-of-function mutations in the STAT1 gene^24^. This observation suggests that JAK inhibitors, which have been successfully used to treat some inflammatory complications of STAT1 GOF patients^52^, may also be a therapeutic option for inflammatory complications of CGD. While IFN signatures have been reported in some inflammatory conditions^53,54^, their presence and relative magnitude have not been comparatively analyzed across multiple monogenic disorders. These observations and hypotheses highlight the power of the comparative approach taken to study monogenic diseases in this study.

Our bottom-up analysis of subject-level immune states revealed an axis (jPC1) of natural subject-to-subject variation captured by both blood transcriptomic and circulating protein data. Surprisingly, this was not driven by differences among diseases or between healthy and sick patients because a similar, correlated principal axis emerged from the data of sick patients or healthy subjects alone. This axis was also highly concordant with the IHM derived through a supervised machine learning analysis for differentiating healthy from sick patients in our cohort. Thus, the unsupervised and supervised analyses independently converged on a measure of immune health potentially applicable to diverse populations. Supporting this notion, the applicability of the IHM was validated in three independent and biologically distinct datasets. First, we showed that the IHM signature was lower (associated with poor immune health) in patients from a meta-analysis of several polygenic autoimmune and inflammatory diseases. Second, it was associated, when evaluated pre-vaccination, with the antibody response to seasonal influenza vaccination in older individuals, pointing to a potential baseline determinant of vaccine responsiveness in this population. This is notable because the baseline immune statuses of the elderly are often highly heterogeneous and shaped by myriad complex factors (e.g., medications and comorbidities)^41,55^. Finally, it was negatively correlated with age in healthy subjects in our cohort and in a large independent cohort of healthy adults age ~20-90, consistent with the expectation that immune health declines with age. The IHM is based on a relatively small number of parameters and can be evaluated using circulating proteins from serum alone, and thus can potentially serve as an inexpensive tool for monitoring immune states and functions in diverse populations.

Given the applicability of the IHM in a range of biological scenarios, it is perhaps not surprising that IHM transcriptional scores appeared lower in nearly every peripheral immune cell type from patients with various polygenic or idiopathic immunological diseases. This coherent signature could be, at least partly, driven by cell-extrinsic factors, such as some of cytokines (interferons) and tissue growth/homeostatic factors (e.g., Neurotrophin-3) revealed by the IHM circulating protein correlate analysis. This result obtained using another independent dataset further validates the notion that the IHM likely has applicability beyond the monogenic conditions explored in this study. Interestingly, these coherent IHM signals across cell types were seen in only a subset of cell types when assessing the cell type specific correlation between the IHM transcriptional score and age in healthy subjects, including LDGs and some regulatory and effector memory T-cell subsets. LDGs (which includes low density neutrophils) and these T-cell subsets have been implicated in a spectrum of immunological and inflammatory conditions, including autoimmunity, cancer, and cardiovascular disease^56-59^. The age-related signals that we detected in Tregs and neutrophils confirm previous reports that aging contributes to their pathologic potential^56,60^.

Markers of systemic inflammation (e.g., CRP and serum amyloid A), RDW, and NK cell frequencies were some of the key constituents of the IHM. RDW and inflammatory markers were negative indicators of immune health. Increased RDW has been associated with human aging and several pathologies, including heart disease and cancer^19^, as well as mortality and morbidity risks (e.g., in Coronavirus Disease 2019^61^). While the mechanisms behind these associations are not entirely clear, increased RDW is known to reflect dysregulation of erythropoiesis and potential reductions in the rate of RBC turnover^18,62^. Conversely, higher NK cell numbers were associated with higher IHM scores. Aging, which is associated with the IHM in our study, is known to be associated with decreased NK cell production in the bone marrow. While it is unclear whether decreased bone marrow output or reduced expansion capacity of specific NK cell subsets played a role in the lower NK cell numbers we observed across multiple diseases, the association of both RDW and NK cell frequency with the IHM suggests that disruption of hematologic homeostasis may be involved.

Inflammaging (chronic, sterile inflammation that increases with age) has been linked to age-related adverse outcomes such as cardiovascular disease. However, the inflammatory mechanisms or molecules responsible have not been well characterized^37,44,63^. Inflammaging has been linked to increased IL-6 in the literature, although there has been conflicting data^63^; IL-6 was neither correlated with the IHM in our study nor a key feature of an inflammatory aging (iAge) “clock” recently developed from ~1000 healthy individuals^45^. That study identified CXCL9/MIG as an informative feature of age-related inflammation. In our data, CXCL9 is a member of the protein module PM2, a key component of the IHM. PM2 also includes other inflammatory cytokines (e.g., IL-23) and IFN-related or -induced proteins (e.g., IP-10/CXCL10, I-TAC/CXCL11). As expected, the IHM was negatively correlated with CXCL9/MIG, but it remained correlated with age even when CXCL9/MIG and PM2 were removed, consistent with our findings that the protein IP-10/CXCL10 was negatively correlated with the IHM independent of age in healthy individuals only. More broadly, the IHM (and jPC1) was surprisingly variable even among apparently healthy subjects; the correlation between circulating proteins (including both negative and positive indicators of immune health) and the IHM in healthy subjects is also independent of age, suggesting that the IHM captures aspects of immune health not linked to age and inflammaging. Thus, the IHM, as measured by easy-to-assay serum protein parameters for example, could be applicable to the healthy population.

It has been recognized that despite ample clinical tools for assessing general physiologic and organ system function and health (e.g., cardiovascular function), aside from the CBC, such tools are largely missing for the immune system^11,64^. This is partly because the function and pathology of the immune system are wide ranging and thus unified definitions and metrics of general immunological health have been elusive^11,65,66^. Here we have developed a framework for defining and quantifying immune health by searching for personal, temporally stable immune parameters enriched in health (i.e., in healthy subjects) but depleted in patients across diverse pathologies due to perturbations of normal immune functions. The resulting measure was surprisingly generalizable to different patient populations and healthy individuals. Further refinement and development of such approaches, e.g., by increasing the diversity and number of studied subjects including the incorporation of additional pathologies, utilizing measurements from tissues, and modeling potential modifiers such as sex and genetic factors, hold promise for the development of clinically useful immune health monitoring tools to advance personalized and preventative medicine^67,68^.

### Limitations of the Study

As expected, some of the observed immune variations across individuals in our cohort are reflected by information shared across correlated data modalities (e.g., circulating proteins, whole blood transcripts, and cell frequencies); however, all major results presented were robust to variations in circulating immune cell frequencies and still significant when controlling explicitly for cell-frequencies. Our analysis of temporal stability by estimating between-subject variations was limited by a relatively small number of patients with repeat samples. Despite this we observed consistent temporally stable, between-subject variations among data modalities, including cellular, transcriptomic, and circulating protein parameters, that dominate relative to those attributable to disease condition, medication, age, and sex; these results are also robust to resampling noise as suggested by Jackknifing analysis. Although achieving mechanistic insights into any specific monogenic disease was not our goal, we demonstrated how this multimodal data could be used to yield new observations and hypotheses concerning disease etiology and therapeutic targets. For example, through our comparative study of interferon-related transcriptional signatures among several diseases, we were able to suggest JAK inhibitors as a possible therapeutic to further explore for CGD. Lastly, some of the major signals related to the IHM may partially reflect age-related decline of immune health and increase in inflammation in healthy individuals^69^. However, even when we examined the jPCs, which represent principal components of variation shared by the transcriptomic and serum protein data, there was considerable variation unexplained by age. Furthermore, similar positive and negative circulating protein correlates of the IHM emerged regardless of whether age was included as a co-variate. Thus, our work provides a broadly useful dataset and a conceptual framework and markers for defining and measuring human immune health.

## Methods

### Patient population and sample collection

Samples were collected on patients with monogenic immune disorders enrolled on National Institutes of Health (NIH) protocols 00-I-0159 (NCT00006150), 01-I-0202 (NCT00018044), 07-I-0033 (NCT00404560), 13-I-0157 (NCT01905826), 93-I-0119 (NCT05104723), 04-H-0012 (NCT00071045), and 94-HG-0105 (NCT00001373). Samples were collected when patients presented to NIH for inpatient or routine outpatient care between September, 2015 and November, 2017. Samples from matching healthy subjects were collected from subjects enrolled on NIH protocols 91-I-0140 (NCT00001281) and 15-I-0162 (NCT02504853). These studies were approved by the NIH Institutional Review Board and complied with all relevant ethical regulations. Informed consent was obtained from all participants.

### RNA isolation

Blood was drawn directly into the Tempus Blood RNA Tube (Thermo Fisher Scientific, Waltham, MA) according to manufacturer’s protocol. Two Tempus tubes were collected per patient and healthy donor. The blood sample from each Tempus tube was aliquoted in to two 4.5mL cryovials. These cryovials were directly stored in −80°C freezer for long term.

RNA was isolated from tempus blood samples using the Tempus Spin RNA Isolation kit (Thermo Fisher Scientific, Waltham, MA) with following modifications to the manufacturer’s protocol: For each sample, 4ml of tempus blood sample was added to a 50ml conical tube containing 1.5ml of 1x PBS. The tubes were vortexed at full speed for 30 seconds, followed by centrifugation at 3000 g for 1 hour at 4°C. After centrifugation, the supernatant from the tubes was decanted and tubes were placed upside down on clean paper towels for 2 minutes. 400ul of RNA Purification buffer was added, vortexed briefly to resuspend the pellet and transferred the RNA to a purification filter with a pre-wet purification filter with 100ul wash solution I. The tubes were centrifuged at 16,000 g for 30 seconds and liquid waste was discarded. A second wash was done with 500ul wash solution I, followed by centrifugation at 16,000g for 30 seconds. The filter was washed with 500ul of wash solution 2 and centrifuged at 16,000 g for 60 seconds. DNase treatment was performed by adding 100ul of AbsoluteRNA wash solution (Thermo Fisher Scientific, Waltham, MA), followed by 15 mins of incubation at room temperature and 5 mins of incubation with wash solution 2. The tubes were spun at 16,000 for 60 seconds. The liquid waste was discarded, and empty tube was spun at 16,000 g for 30 seconds to remove any residual liquid and the filter was inserted into a new collection tube.

The Nucleic Acid Purification Elution Solution was pre-warmed at 45°C. 100ul of this pre-warmed elution solution was added to the filter and incubated at 37°C for 5 minutes. The tubes were spun at 16,000 g for 2 minutes. The eluate was pipetted back to the filter and spun again at 16,000 g for 1 minutes such that the eluate was collected in a new collection tube. 90ul of the eluate was transferred to a new tube.

RNA QC was performed using Qubit RNA BR assay (Thermo Fisher Scientific, Waltham, MA) and Agilent RNA (Agilent Technologies, Santa Clara, CA). The average RIN was 8.26 and average yield was 4.69 μg for the RNA samples.

### Serum isolation

Serum was collected directly in Serum Separator Tubes and allowed to clot at room temperature for a minimum of 30 minutes. Within two hours of blood collection, the tubes were spun at 1800 g for 10 minutes at room temperature. The top (serum) layer was removed via pipette and stored in individual vials at −80°C.

### Microarray hybridization

All blood samples at different time points from the same subject were processed together. Before assay, 396 samples were carefully batched into 14 groups according to their age, gender and race but run blindly. One in-house reference sample was simultaneously processed with the real samples in each batch. RNA was amplified from 300 ng of total RNA using Ambion WT Expression Kit (Thermo Scientific, Wilmington, DE). Fragmented single-stranded sense cDNA was terminally biotinylated and hybridized to the Affymetrix Human Gene 1.0 ST Arrays with the probes for 36,079 RefSeq coding and noncoding transcripts and 466 lncRNA transcripts (Affymetrix, Santa Clara, CA). The arrays were then washed and stained on a GeneChip Fluidics Station 450 (Affymetrix); scanning was carried out with the GeneChip Scanner 3000 and image analyzed with the Affymetrix GeneChip Command Console (AGCC) software 4.0.

### Somalogic SOMAScan Blood proteomic assays

Proteomic profiles for 1,305 SOMAmers in serum were assessed using the 1.3K SOMAscan assay at the Trans-NIH Center for Human Immunology and Autoimmunity, and Inflammation (CHI), National Institute of Allergy and Infectious Disease, National Institutes of Health (Bethesda, MD, USA). Samples were run according to Somalogic standard operating procedures. If operators identified presence of hemolysis in sample, those were marked for presence of hemolysis (1 low- 4 high). In addition to Somalogic quality control samples, internal QC of the runs (cross checked of hemolyzed samples and outliers) was performed using CHI webtools (Cheung *et al*). A total of 358 samples were included in this analysis. Two samples with high levels of hemolysis (hemolysis score 4) and one sample with odd appearance were removed from downstream analysis resulting in 355 total samples. The SOMAscan assay has a total of 1322 SOMAmer Reagents, and of these 12 are hybridization controls, which were removed after hybridization normalization. 5 are nonspecifically-targeted SOMAmers (P05186; ALPL, P09871; C1S, Q14126;DSG2, Q93038; TNFRSF25, Q9NQC3; RTN4, P00533; EGFRvIII, leaving 1305 somamers targeting 1273 unique proteins. The protein panel includes 4 proteins that are rat homologues (P05413; FABP3, P48788; TINNI2, P19429; TINNI3, P01160; NPPA) of human proteins and 4 viral proteins (HPV type 16, HPV type 18, isolate BEN, isolate LW123).

### Somalogic normalization

The Somalogic SOMAscan 1.3k assay data was normalized using the procedure outlined in^1^ followed by additional inter-plate batch correction prior to log transformation. As described in^1^, hybridization control normalization (HybNorm) was first performed for each well on a plate, and subsequent inter-plate calibration (CalNorm) was used to correct for plate-specific effects between plates sharing the same Somalogic control samples. After these steps, median signal normalization was performed on each group of samples from Somalogic plates that used the same Somalogic control. This median normalization was performed to correct for shifts in the median somamer RFUs across samples that may have been due to technical effects rather than biological ones.

Additionally, four bridge samples (QC_CHI), derived from healthy donor blood, were added to every run to allow in-house batch calibration normalization. These QC_CHI samples were mixed pools of serum samples of healthy donors from the Center for Human Immunology. In each batch, the QC_CHI controls were used for inter-plate calibration after the initial inter-plate calibration with the Somalogic control samples. After this step, all relative protein expression values were log_2_ transformed.

### Curation of patient medication and medical metadata

Patient medical records were evaluated at the level of individual patient visits by trained medical personnel. Medications used at the time of the visit were documented based on notes from that visit; at the time of entry, medications were matched to the closest corresponding term in MeSH. Medications were documented to include the route, dose, frequency, potency (when applicable), date started and date ended (when available). Medical conditions were obtained from chart review and were documented to include past and current medical history. The conditions were entered by hand into a SQL database and selected from available terms in the Human Phenotype Ontology (HPO). Conditions that were unable to be reasonably matched to HPO terms were entered with free text. Current medical conditions were denoted as one of four options: 1) acute, active; 2) acute, resolved; 3) chronic, flare; 4) chronic, stable; 5) future (for planned procedures or therapies).

### Microarray normalization, processing, filtering

Data were normalized and summarized to the probeset level using the RMA algorithm implemented in the oligo R package^2^. Probesets mapping to multiple genes were discarded. To select a single probeset for each gene, principal components analysis was performed for every group of probesets corresponding to a given gene. The probeset most correlated with the first principal component of this group was chosen as the “best” probeset to represent the expression of this gene. With the microarray data summarized to the gene level, genes were then filtered to remove genes that appeared lowly expressed or showed higher technical variation than biological variation. Lowly expressed genes were identified as discussed in^3^; briefly, a histogram of the median log2 expression values were plotted and a lowly expressed local maximum was identified. There exists a “plateau” where genes with low median intensity are enriched. A manual threshold was selected to remove all genes in this enriched low intensity area of the histogram. To determine the relative amounts of biological vs technical variation, the variance of a gene in technical control samples (identical runs of same RNA) was compared to the variance of the gene across all of the patient/healthy control samples. Those genes with higher variance in the technical controls were removed from further analysis.

### Complete Blood Counts and lymphocyte phenotyping

Subjects had standard complete blood counts (CBCs) performed at the NIH Clinical Center in the Department of Laboratory Medicine. Lymphocyte (T cell, B cell, NK cell) flow cytometry quantification was performed using the BD FACS Cantoll flow cytometer. The following parameters were collected on most patients, but were removed in downstream analysis for the given reasons:

- Hematocrit measurements were removed, as they are highly redundant with hemoglobin measurements
- Nucleated red blood cell measurements were removed, as they were zero for the majority of patients.
- MPV, immature granulocytes (concentration and percent WBCs), CRP, and ESR measurements were removed, as they were missing for 14, 62, 53, and 61 samples respectively.

Three samples were removed due to inconsistencies found in their data (the sum of the absolute counts of cells from the TBNK assay was highly inconsistent with the total lymphocytes from the complete blood counts).

Absolute counts of leukocytes (including TBNK) were used for downstream analysis. The neutrophil to lymphocyte ratio (NLR), the ratio between the neutrophil absolute counts and lymphocyte absolute counts, was included as an additional CBC parameter for classification due to its previously described association with multiple medical conditions such as infections and cancer^4,5^.

### Assignment of subjects to the main and set-aside cohorts

From a total of 270 subjects (including 42 healthy controls), two sub-cohorts, namely *main* and *set-aside*, were created with the purpose of holding out the *set-aside* group for future validation and testing of specific hypotheses. Subjects with multiple visits were assigned to the main group to allow for the assessment of temporal, intra-subject stability. The rest of the participants were randomly assigned to one of the sub-cohorts to achieve a ratio of approximately 80% *main* to 20% *set-aside* for each of the conditions, resulting in 217 and 53 subjects in the two groups, respectively. All analyses unless explicitly stated utilize only the *main* subjects.

### Averaging of technical replicate samples

Each measured parameter among technical replicate samples (samples taken from a patient during the same visit) were averaged for downstream analysis after normalization (including log2 transformation for the Somalogic and Microarray data). Samples from the same visit were considered technical replicates, although a visit could be an inpatient visit spanning several days or a one-day outpatient visit (of 364 total visits in the study, 7 visits consisted of blood draws over multiple days and 6 consisted of multiple draws on the same day). This was done for gene log-intensities, protein log-RFUs, and CBC parameters. We refer to the data after averaging across technical replicates as “sample-level” data.

### Averaging of biological replicate samples

In situations where we wished to investigate data at the subject level rather than sample level, we averaged each parameter over biological replicates in the sample-level data. We refer to the result as “subject-level” data. Note that patient ages associated with a subject for a data type were assigned to be the average age across all visits for which a sample of that data type was collected. The largest time difference between samples from the same subject was 369 days.

### Gene and protein module creation

Weighted Gene Correlation Network Analysis (WGCNA)^6^ was used to form modules of genes and modules of proteins using the subject-level data (see averaging of biological replicate samples). The parameters chosen were the same as the tutorial available at https://horvath.genetics.ucla.edu/html/CoexpressionNetwork/Rpackages/WGCNA/Tutorials/FemaleLiver-02-networkConstr-man.pdf with the following deviations: for the microarray data and Somalogic data, a soft-threshold of 12 was manually chosen. Additionally, for the Somalogic data the *cutreeDynamic* method parameter was set to ‘tree,’ as this provided modules with greater variation explained by the 1st principal component compared to the ‘hybrid’ method, as used in the microarray WGCNA analysis.

Prior to module creation with WGCNA, samples were flagged as outliers by cutting an agglomerative hierarchical tree formed from distances between samples in the sample-level data. Data were scaled to unit variance prior to distance calculation. This was done separately for each data type and tree cut heights for the proteomic and transcriptomic data hierarchical trees were manually chosen 75 and 250 in each data type respectively. For both data types, the minimum branch size required so that samples on the branch were not removed was set to 10. The subject-level data was then rederived by averaging as before, but without these outlier samples. Although outliers were removed during the module creation process to avoid these extreme samples creating undue impact on the modules, these samples were included for downstream analyses, as they may have been flagged as outliers due to their extreme phenotypes (e.g. marrow failure) rather than technical noise. Thus, module activity scores were still computed for these outlier samples, even though they were not used to inform the creation of the modules.

### Gene and protein module activity scores

Module activity scores (sometimes referred to as module eigengenes) for a gene or protein module were calculated for each sample in the following way: First, the subject-level data was recomputed (using the same procedure described in ‘Averaging of biological replicate samples’) from the sample-level data, after removing the outlier samples in the given data type. Next, the module’s first principal component axis (PC1) was found through performing PCA on the recomputed subject-level data, subsetted to only include features belonging to the module. Then, for each sample in the sample-level data (including the outliers not used when deriving the modules and principal component axes), the projection of the sample’s feature vector, subsetted to only the features in a given module, onto the PC1 for that module was computed. This result was assigned to be that sample’s activity score for that module. As the modules were derived through signed WGCNA, the features in the modules were designed to be positively correlated with one another; however, PCA can produce PC’s that are positively or negatively correlated with the features. If a module’s activity scores were negatively correlated with more features in the module than were positively correlated, we multiplied that module’s activity scores (derived via PC1) by −1, such that the scores were positively correlated with most of the features in the module. Samples were not assigned a module activity score for the grey WGCNA module.

### Analysis of feature stability

Variance component models were fit using the variancePartition package^7^ to estimate the sources of variation from a list of covariates for each feature in the transcriptomic and proteomic data, leveraging repeat samples to estimate intra-subject temporal variation in parameters. Two variance partition models were fit; The first model (VP_M1) only includes the subject as a random effect, with all other variation being considered “residual.” The second model (VP_M2) includes subject, condition, and various binary medication variables as random effects. The medication groups included in VP_M2 were Monoclonal antibodies(not including those for TNF and IL1), Anti-fungal, Antibiotic, Anti-TNF, Anti-IL1, Anti-inflammatory, IgG-replacement, IFN-gamma, Immune-stimulator, Immunosuppressant, and Steroids. As patients often were taking different combinations of medications, which potentially changed between repeat samples, the medications were coded as binary variables denoting whether a patient was or was not taking a given medication at the time of sampling. The individual variance contributions assigned to each of the medications were then summed to a single medication-associated variance contribution. Medications were included in the model if they were used by many patients and not highly confounded with one of the condition groups.

A feature was deemed to be stable if VP_M1 estimated that there was more intrer-subject variation than intra-subject variation in that feature (i.e. 50% or greater of the variation is explained by patient covariate in VP_M1). This determination was made for all data types (transcriptomic measurements, transcriptomic modules activities, proteomic measurements, proteomic module activities, and CBC+TBNK parameters). In various downstream analyses, only the stable features as determined by this method were used.

To evaluate the robustness of these estimates, VP_M1 was performed with 100 replicates of jackknife resampling in which 80% of subjects with repeats and 80% of subjects without repeats were selected. Results were summarized with the mean variance explained by subject across jackknife samples and the 95% confidence interval was taken as 2.5% quantile and the 97.% quantile across jackknife samples.

### Disease Signature/Differential expression analyses

To determine the disease signatures, Limma^8^ was used to fit linear models and test differential expression for each feature (Somamer, transcript, module or CBC/TBNK parameter). A single model was fit for each feature that accounted for Condition, Gender, and Age, and Visit Type (whether or not the patient reports feeling sick on a given visit): feature ~ condition + age + gender + visit_type.

T-statistics and p-values were computed for the following contrasts of the coefficients:

- Disease vs. Healthy signatures
  - Healthy was coded as the reference level and a t-statistics were computed for the coefficient for each condition
- Each disease vs. all other diseases
  - A contrast matrix was made such that each disease was compared to all other diseases (the weights for each ‘other’ disease group were set to be equal).
- Comparison-specific contrasts were created to compare single diseases to others or groups of diseases to other groups.

For tests involving the gene expression or proteomic modules, standard t-statistics (those computed without empirical bayes moderation) were used to compute p-values due to the lower number of features. For the individual proteomic or transcriptomic features, the empirical Bayes moderated t-statistics ^8^ were computed and used to compute p values. Multiple hypothesis correction was performed using the Benjamini-Hochberg^9^ method to compute FDR-adjusted p values.

### Clustering genes within TM1: Interferon

The genes with TM1: Interferon were subclustered by computing the Euclidean distance matrix between all genes based on the T-statistics from the differential expression analysis comparing all conditions to Healthy Controls. The genes were clustered using Ward’s method (method = “Ward.D2”) with the hclust function in R. The hierarchical clustering tree was then cut to produce three clusters with the cutree function with k = 3.

### JIVE analysis

The whole blood microarray and serum proteomics data (Somalogic) were filtered to select only stable features (see *determining feature stability*). Data were averaged to the subject level (see averaging of biological replicates). The JIVE algorithm^10^ was then used to partition the data into joint (sharing axes variation between the transcriptomic and proteomic data) and individual (unique to a data-type) components. Input data were first z-score normalized for each feature and then each input matrix was scaled by the frobenius norm of that data type so as to not give greater weight to data with more features (i.e. the transcriptomic data). The JIVE algorithm produces 3 matrices for each data-type, representing joint (shared between data types), individual (unique to that data-type) and residual (potentially noise) variation. JIVE PC scores were computed for each subject using the prcomp function from R, using the resulting joint, and individual matrices as inputs. To compute the joint PC scores (jPC’s), the transcriptomic and proteomic joint matrices were concatenated to a single joint matrix prior to calculation of the PC scores.

### JIVE variance explained calculations

To calculate the amount of variation explained by each of the joint and individual components from the JIVE analysis, we computed the frobenius norm of the input data (proteomic or transcriptomic) to determine the total amount of variation present in a given data matrix. This same computation was then applied to the resulting joint and individual matrices. Dividing the variation in the joint and individual matrices by the amount of total variation gives the variance explained by each of these respectively. Lastly to determine residual variation, the joint and individual variation were subtracted from the total variation.

### JIVE PC geneset enrichment

To determine the gene set enrichments for the JIVE PC’s, the whole blood microarray and serum proteome data were separately correlated with each JIVE PC. Genesets were then tested for enrichment of correlation to each PC in each data type separately, using the two-sided t-test with correlation described in Wu, Di, and Gordon K. Smyth. Nucleic acids research 40.17 (2012), using the cameraPR function from limma ^8^ with use.ranks = FALSE.

### Leukocyte composite score

A leukocyte composite score was computed for each patient by first averaging repeated observations from a given patient. A Z-score was then computed for the lymphocyte, neutrophil and monocyte counts relative to the healthy mean and standard deviation, for that parameter. The three Z-scores were then averaged across the cell-types to give the final composite score.

### Creation of Immune Health Metric

The Immune Health Metric presented represents the likelihood that a given subject is a healthy control according to the leave one out cross validation (LOO CV) prediction probabilities of our random forest model.

Prior to training the models, we subsetted the subjects to those that had measurements from all of the following data: proteomic, transcriptomic, and CBC/TBNK (and passed respective quality checks). Biological replicate samples from the same patients were averaged, so that each subject had one associated value for each measured feature. Features included for classification were subsetted to those for which the VP_M1 variance partition model assigned at least 50% of the variation to the patient covariate (i.e. the stable features).

Three unimodal classifier schemas were designed: a proteomic module classifier, a transcriptomic module classifier, and a CBC parameter classifier, using the stable features from each respective data type.

Two multimodal classifiers were also created: the first included all features from the three unimodal classifiers. The second included all features from the first, but also included the log-RFUs of all singleton proteins (the proteins in the grey Somalogic module). Each classifier described above was then evaluated using leave-one-out cross validation, and an ROC curve was generated from the LOO CV probabilities of being a healthy subject (the positive class).

Predicting healthy subjects vs. disease using all subjects, we computed the LOO CV prediction probabilities that an individual was a healthy control, that we termed the Immune Health Metric.

### Classification accuracy using set aside patients

The second multimodal classifier incorporating module activity scores, immune cell frequencies, and grey module protein RFUs was trained using all subjects in the *main* set of subjects. The disease vs. healthy status of set aside subjects was then predicted and an ROC curve was generated from the predicted probabilities of being a healthy subject (the positive class).

### Statistical testing of classification feature global variable importance

For each classifier, the global variable importance (GVI) of all features were collected after training the classifier on all subjects used in the creation of the Immune Health Metric.

To find the significance of the global variable importance (GVI) for each feature, permutation testing was performed to determine how often the GVI, as estimated by classifiers training on permuted class labels, was higher than the classifier trained on the true labels. A total of 10,000,000 permutations were performed.

### Condition-specific classifiers

One-versus-all-condition binary classifiers were created for the largest groups of patient conditions: CGDs (XCGDs and 47CGDs were combined), Job, STAT1 GOF, and FMF. Each one-versus-all classifiers for each group were created analogously to the multimodal classifier including all modules, CBC +TBNK, and grey module proteins created to differentiate healthy subjects from monogenic patients. Feature GVIs were identified and tested analogously as well. Note that for the disease-versus-all classifiers, healthy controls were excluded from the LOO CV model training, prediction, and calculation of feature GVI.

### Transcriptional surrogate signatures for autoimmunity meta-analysis validation

Transcriptional signatures for features from the three following categories were created:

- Immune Health Metric
- jPC1
- Features: all features from multimodal classifier that passed GVI testing with an FDR-adjusted p value of less than 0.20

Signatures in the indexes and features categories both were formed by taking the 150 genes from the stable microarray features with highest correlation to the feature (based upon correlation with all subjects in our training cohort, including healthy controls). Selected genes were then subsetted to those with a Spearman correlation to the feature of interest of more than 0.35 in magnitude. Genes in the signature were then divided into two groups: those positively correlated with the index/feature of interest, and those negatively correlated. Module signatures were all simply composed of the genes that the module was comprised of (stable and unstable). All these genes were placed in the positive correlates group of the signature, as we used a signed WGCNA performed to derive the modules.

To assign each subject in the validation study a signature score, we subsetted the genes in the surrogate signatures to those also measured in the validation studies and we then averaged the z-scores of each gene/protein (scaled across subjects) for each gene in the signature. Note that z-scores of proteins in the ‘negative correlates’ group were flipped in sign prior to averaging.

### Proteomic Immune Health Metric surrogate signature for aging validation using data from Tanaka 2018

The proteomic IHM surrogate was derived and computed analogously to the transcriptional surrogate signatures as described above, with one small modification: to ensure that the signature was not reliant on proteins that had substantive relative differential abundance in serum compared to plasma (the data in which we planned to test these signatures), we removed any Somamers that fell into different dilution groups between plasma assays and serum assays.

### Autoimmune disease cohort meta-analysis

Comparison group pairs (CGPs) for the OMiCC Jamboree^11^ were used to test our transcriptional surrogate signatures in other data sets. Briefly, CGPs from the same study and platform were combined to ensure that samples were not being replicated across studies. Samples from the same patient in a study were removed manually. Several CGPs used in the OMiCC jamboree were removed for the following reasons:

- CGPs/studies of systemic lupus erythematosus (SLE) appearing in Lau *et al*^11^ were removed as many genes in the signatures to be tested were not present in the platforms used.
- CGP ‘GSE9006-Diabetes_Mellitus,_Type_1-PBMC_newlydiagnosed_paired with 1 month follow up::GSE9006-Healthy-PBMC_unpaired’ was not included because samples in this CGP were follow up samples from another CGP, GSE9006-Diabetes_Mellitus,_Type_1-PBMC_newly diagnosed_unpaired::GSE9006-Healthy-PBMC_unpaired
- CGPs ‘Jam_human_RA_GSE26554-JIA-PBMC::Jam_human_RA_GSE26554-Control-PBMC’, ‘Jam_Human_RA_JIA-PBMC::Jam_Human_RA_Controls-PBMC’, ‘Jam_human_RA_GSE26554-OligoarticularJIA-PBMC::Jam_human_RA_GSE26554-Control-PBMC’, and ‘Jam_Human_RA_JIA-PBMC::Jam_Human_RA_Controls-PBMC’, were removed because the all had many overlapping samples with another CGP already included in our study, Jam_Human_RA_JIA-PBMC::Jam_Human_RA_Controls-PBMC.
- CGP ‘Jam_human_RA_GSE61281-Psoriatric_arthritis-Whole_blood::Cutaneouspsoriasis without arthritis_GSE61281-Cutaneous_psoriasis_without_arthritis-Whole_blood’ was removed because the control patients had psoriasis.

Additionally, some samples were removed within certain studies

- GSE21942
  - GSM545843, GSM545845 were removed as these were technical replicates of other samples in the study
- GSE30210
  - We removed additional biological replicates from patients that were sampled longitudinally and we selected the last sample for each patient
- GSE8650
  - We removed additional biological and technical replicates from the same individual. The last sample was selected for each patient.
  - Samples GSM214490 and GSM214492 were removed as they were believed to have unreliable diagnoses according to the original publication
- GSE15645
  - We removed patients who were experiencing clinical remission of symptoms
- GSE42834
  - We removed patients with non-active sarcoid

A complete listing of the studies and all case/control samples in the meta-analysis can be found in Supplementary Table 19

Each study was quantile normalized within the study. The standard pipeline from the metaIntegrator package^12^ was then used to compute meta effect sizes of each of the surrogate signature scores. Meta-analysis was also performed for all genes that overlapped with those in our the monogenic microarray data and Wilcoxon tests were also used to determine whether genes belonging to each transcriptomic surrogate signature tended to have higher meta-effect sizes than genes that did not belong to the signature.

### Overlap of Baltimore Aging signature and Proteomic Immune Health Metric

We considered the proteins passing an FDR-adjusted significance threshold of 0.05 from Supplementary table 3 of Tanaka *et al*^13^ as the previous aging signature. These proteins were compared to the proteins from the Immune Health metric proteomic surrogate with a one-sided Fisher’s exact test, with the alternative hypothesis being that the overlap was greater than that expected by chance.

### Gene set enrichment analyses

Gene modules from the transcriptomics data were tested using hypergeometric tests for the following collections of gene sets: The Li blood transcriptomic modules^14^, Kyoto Encyclopedia of Genes and Genomes^15^, Reactome^16^, and Gene Ontology Biological Processes^17^. For each module, FDR multiple hypothesis corrections were performed on all gene sets (pooled across collections).

Proteomic modules were tested for gene set enrichments analogously after converting each protein targets of Somamers to their respective gene according to the SomaScan assay. Proteins that mapped to multiple genes were removed from the analysis. Additionally, some genes corresponded to multiple proteins. In this case, when testing a gene module, genes that mapped to both proteins in and outside of the module were removed from the module and the background proteins.

An analogous analysis was performed for the proteomic modules using gene sets from the Human Protein Atlas^18^. Gene sets were made for various tissues by looking for proteins enriched for that tissue based on the HPA. The following categories were considered for enrichments: “enriched”, “enhanced”, and “tissue enriched.”

### Correlation of serum proteins with IHM surrogate transcriptional signature

The correlation, without removing the effect of age, was computed simply by computing the Spearman correlation of every protein with the IHM surrogate signature. We additionally computed partial correlations where the effect of age had been removed from both the protein data and IHM transcriptional surrogate signature by using the limma removeBatchEffect function with age as the single covariate, which fits a linear model (feature ~age) to remove the effect of age prior to computing the correlation of each protein with the IHM transcriptional signature.

### Testing IHM and jPC1 signatures in Ota *et al*^19^ 2021 sorted cell data

Data were downloaded from https://ddbj.nig.ac.jp/public/ddbj_database/gea/experiment/E-GEAD-000/E-GEAD-397/. For each cell-type, the log cpm values with TMM normalization were computed using edgeR. We noted a large batch effect due to the “Phase” of the study and thus removed the phase effect at the individual gene level using limma’s removeBatchEffect function. After this, genes were z-scored normalized and signature scores were computed as described in the section above Transcriptional surrogate signatures for autoimmunity meta-analysis validation. We then tested for differences in signature scores between healthy and disease using linear models with limma. The association with age within healthy individuals only was assessed using the Pearson correlation as implemented in the cor.test function in R.

### Vaccination response in elderly meta-analysis

**Table:** 

Cohort	Ages	Source
Stanford (2009-2010)	61-90 years	Furman, 2013 (SDY 212)
Yale (2011-2012)	66-93 years	Avey, 2020 (GSE65442)
Yale (2012-2013)	65-88 years	Avey, 2020 (GSE95584)
Yale (2013-2014)	65-86 years	Avey, 2020 (GSE101709)

Gene expression profiles for Yale vaccination subjects were quantile normalized using the R package *limma*. Processed expression data from SDY212 was downloaded from *ImmuneSpace*. Each dataset was filtered to baseline, pre-vaccination samples from subjects over the age of 60. High and low antibody response labels for each subject were derived from HAI titer measurements using the maximum residual after baseline adjustment (maxRBA) end point ^20^. IHM signature scores were calculated in each subject using the *MetaIntegrator* R package. Briefly, the signature score for each subject was calculated from normalized, log2 transformed gene expression data by taking the geometric mean of positive signature genes and subtracting the geometric mean of negative signature genes. The standardized mean difference of baseline IHM scores between high and low antibody responders was estimated by fitting a random effects model using the *metafor* R package.

### Checks of robustness to variation in cell frequencies

Linear models were fit using the lm function in R both with and without including cell frequencies in the model. Cell frequencies were included as percent of total white blood cells and included major cell populations from the CBC/TBNK, specifically neutrophils, monocytes, CD4 T-cells, CD8 T-cells, B cells, NK cells, eosinophils, and basophils. The percent mediation, which reflects how much of the main effect can be explained by additional covariates, was calculated as: 1 – coefficient_without_controlling_for_cell_freq / coefficient_with_controlling_for_cell_freq.

## Extended Data

**Extended Data Table 1.** Description of monogenic diseases in this study.

Autoinflammatory Diseases							
Disease Acronym	Gene/Protein	Disease Name	OMIM Number	Inheritance; Mutation effect	Phenotype	Pathomechanism of Inflammation	Ref
**CAPS**	*NLRP3* / NLRP3	Familial cold autoinflammatory syndrome (FCAS): NLRP3-associated autoinflammatory disease-mild Muckle-Wells syndrome (MWS): NLRP3-associated autoinflammatory disease-moderate	120100, 191900	Autosomal Dominant / De novo; Gain of Function Mutations	Fever, urticaria-like rash, CNS inflammation, bone overgrowth	Constitutively active NLRP3 inflammasome and increased IL-1β production	(Aksentijevich and Schnappauf, 2021; Manthiram et al., 2017; Tangye et al., 2020)
**DADA2**	*ADA2/CECR1* / ADA2	Deficiency of Adenosine Deaminase 2	615688	Autosomal Recessive; Loss of Function Mutations	Fever, lacunar strokes, livedo, immunodeficiency, anemia	Decrease in protein expression/activity leads to preferential differentiation of M1 proinflammatory macrophages,	(Aksentijevich and Schnappauf, 2021; Meyts and Aksentijevich, 2018)
**FMF**	*MEFV* / Pyrin	Familai Mediterranean Fever	249100	Autosomal Recessive; Gain of Function Mutations	Fever, serositis, rash, SAA amyloidosis	Facilitated activation of pyrin inflammasome leads to increased IL-1β production	(Aksentijevich and Schnappauf, 2021; Manthiram et al., 2017)
**HIDS/MKD**	*MVK* / MVK	Hyperimmunoglobulinemia D syndrome / Mevalonate Kinase Deficiency	260920, 610377	Autosomal Recessive; Loss of Function Mutations	Fever, serositis, rash, lymphadenopathy	Decrease in MVK activity enhances IL-1β production through activation of pyrin inflammasome	(Aksentijevich and Schnappauf, 2021; Manthiram et al., 2017)
**PAPA**	*PSTPIP1* / PSTPIP1	Pyogenic Arthritis, Pyoderma Gangrenosum and Acne Syndrome	604416	Autosomal Dominant / De novo; Not known	Pyoderma, pyogenic arthritis, severe cystic acne	Increased affinity to pyrin causes enhanced IL-1β production	(Aksentijevich and Schnappauf, 2021; Manthiram et al., 2017; Tangye et al., 2020)
**TRAPS**	*TNFRSF1A* / TNFR1	TNFR1-associated Periodic Syndrome	142680	Autosomal Dominant / De novo; Not known	Fever, serositis, rash, myalgia, orbital inflammation, SAA amyloidosis	Misfolding of extracellular domain of the receptor leads to intracellular protein retention and increased endoplasmic reticulum (ER) stress	(Cudrici et al., 2020; Tangye et al., 2020)

Primary Immunodeficiency Diseases (see Tangye et al., 2020 for additional phenotypic and functional details and references)						
Disease Acronym	Gene/Protein	Disease Name	OMIM Number	Inheritance; Mutation effect	Phenotype	Ref
**STAT1 GOF**	*STAT1* / STAT1	STAT1-gain-of-function	614162	Autosomal Dominant / De novo; Gain of Function Mutations	Chronic mucocutaneous candidiasis, bacterial infections, viral infections, autoimmunity	(Tangye et al., 2020; Toubiana et al., 2016)
**GATA2**	*GATA2* / GATA2	GATA2 deficiency / GATA2 haploinsufficiency	614172	Autosomal Dominant / De novo; Loss of Function Mutations	Lymphopenia, monocytopenia, myelodysplastic syndrome/acute myeloid leukemia, viral infections, NTM infection	(Spinner et al., 2014; Tangye et al., 2020)
**APDS1**	*PIK3CD* / p110δ catalytic subunit of PI3Kδ	Activated PI3K delta syndrome 1	615513	Autosomal Dominant / De novo; Gain of Function Mutations	Bacterial infection, lymphoproliferation, herpesvirus infections, autoimmunity	(Coulter et al., 2017; Tangye et al., 2020)
**X-CGD**	*CYBB* / p91^phox^	X-linked chronic granulomatous disease	306400	X-linked recessive; Loss of Function Mutations	Bacterial infection, invasive fungal infection, colitis, inflammatory lung disease, autoimmunity	(Arnold and Heimall, 2017; Henrickson et al., 2018; Tangye et al., 2020)
**p47-CGD**	*NCF1* / p47^phox^	Autosomal recessive chronic granulomatous disease due to p47^phox^ deficiency	233700	Autosomal Recessive; Loss of Function Mutations	Bacterial infection, invasive fungal infection, colitis, inflammatory lung disease, autoimmunity	(Arnold and Heimall, 2017; Henrickson et al., 2018; Tangye et al., 2020)
**CTLA4**	*CTLA4* / CTLA4	CTLA4 haploinsufficiency	616100	Autosomal Dominant / De novo; Loss of Function Mutations	Hypogammaglobulinemia, lymphoproliferation, pulmonary infections, autoimmune cytopenias	(Schwab et al., 2018; Tangye et al., 2020)
**PGM3**	*PGM3* / PGM3	PGM3 deficiency	615816	Autosomal Recessive; Loss of Function Mutations	Bacterial infections, atopic dermatitis, elevated serum IgE, skeletal abnormalities, developmental delay	(Bergerson and Freeman, 2019; Tangye et al., 2020)
**LAD1**	*ITGB2* / integrin subunit β2	Leukocyte Adhesion Deficiency type 1	116920	Autosomal Recessive; Loss of Function Mutations	Periodontitis, skin infections, delayed umbilical cord separation	(Almarza Novoa et al., 2018; Tangye et al., 2020)
**IL12R**	*IL12Rβ1* / IL12Rβ1	IL-12 receptor β1 deficiency	614891	Autosomal Recessive; Loss of Function Mutations	Invasive mycobacterial disease, chronic mucocutaneous candidiasis, *Salmonella* infection	(Bustamante et al., 2014; Tangye et al., 2020)
**CARD14 DN**	*CARD14* / Caspase recruitment domain-containing protein 14	Dominant-negative CARD14 deficiency	607211	Autosomal Dominant / De novo; Dominant Negative Mutations	Severe atopic dermatitis, elevated serum IgE, food allergy, asthma	(Peled et al., 2019)
**NEMO**	*IKBKG* / inhibitor of nuclear factor kappa B kinase regulatory subunit gamma	NEMO deficiency	300636	X-linked recessive; Loss of Function Mutations	Ectodermal dysplasia, bacterial, viral, and mycobacterial infections, conical teeth, colitis	(Miot et al., 2017; Tangye et al., 2020)
**STAT3 DN**	*STAT3* / STAT3	STAT3-dominant-negative hyper-IgE syndrome / autosomal dominant hyper-IgE syndrome / Job’s syndrome	147060	Autosomal Dominant / De novo; Dominant Negative Mutations	Bacterial infections, viral infections, atopic dermatitis, elevated serum IgE, skeletal and vascular abnormalities	(Bergerson and Freeman, 2019; Tangye et al., 2020)

Telomere disorders						
Disease Acronym	Gene/Protein or RNA	Disease Name	OMIM Number	Inheritance; Mutation effect	Phenotype	Ref
**TERT TERC**	*TERT* / TERT protein *TERC* / TERC RNA molecule	Telomere biology disorder, or telomereopathy	614742, 614743	Autosomal Recessive; Loss of Function Mutations	Hypocellular and aplastic anemia, pulmonary fibrosis, liver disease	(Townsley et al., 2014)

ReferencesAksentijevichI., and SchnappaufO. (2021). Molecular mechanisms of phenotypic variability in monogenic autoinflammatory diseases. Nat. Rev. Rheumatol.
17, 405–425.3403553410.1038/s41584-021-00614-1Almarza NovoaE., KasbekarS., ThrasherA.J., KohnD.B., SevillaJ., NguyenT., SchwartzJ.D., and BuerenJ.A. (2018). Leukocyte adhesion deficiency-I: A comprehensive review of all published cases. J. Allergy Clin. Immunol. Pract.
6, 1418–1420.e10.2937107110.1016/j.jaip.2017.12.008ArnoldD.E., and HeimallJ.R. (2017). A Review of Chronic Granulomatous Disease. Adv. Ther.
34, 2543–2557.2916814410.1007/s12325-017-0636-2PMC5709447BergersonJ.R.E., and FreemanA.F. (2019). An Update on Syndromes with a Hyper-IgE Phenotype. Immunol. Allergy Clin. North Am.
39, 49–61.3046677210.1016/j.iac.2018.08.007BustamanteJ., Boisson-DupuisS., AbelL., and CasanovaJ.-L. (2014). Mendelian susceptibility to mycobacterial disease: Genetic, immunological, and clinical features of inborn errors of IFN-γ immunity. Semin. Immunol.
26, 454–470.2545322510.1016/j.smim.2014.09.008PMC4357480CoulterT.I., ChandraA., BaconC.M., BabarJ., CurtisJ., ScreatonN., GoodladJ.R., FarmerG., SteeleC.L., LeahyT.R.,  (2017). Clinical spectrum and features of activated phosphoinositide 3-kinase δ syndrome: A large patient cohort study. J. Allergy Clin. Immunol.
139, 597–606.e4.2755545910.1016/j.jaci.2016.06.021PMC5292996CudriciC., DeuitchN., and AksentijevichI. (2020). Revisiting TNF Receptor-Associated Periodic Syndrome (TRAPS): Current Perspectives. Int. J. Mol. Sci.
21, 3263.3238070410.3390/ijms21093263PMC7246474HenricksonS.E., JongcoA.M., ThomsenK.F., GarabedianE.K., and ThomsenI.P. (2018). Noninfectious Manifestations and Complications of Chronic Granulomatous Disease. J. Pediatr. Infect. Dis. Soc.
7, S18–S24.10.1093/jpids/piy014PMC594685829746679ManthiramK., ZhouQ., AksentijevichI., and KastnerD.L. (2017). The monogenic autoinflammatory diseases define new pathways in human innate immunity and inflammation. Nat. Immunol.
18, 832–842.2872272510.1038/ni.3777MeytsI., and AksentijevichI. (2018). Deficiency of Adenosine Deaminase 2 (DADA2): Updates on the Phenotype, Genetics, Pathogenesis, and Treatment. J. Clin. Immunol.
38, 569–578.2995194710.1007/s10875-018-0525-8PMC6061100MiotC., ImaiK., ImaiC., ManciniA.J., KucukZ.Y., KawaiT., NishikomoriR., ItoE., PellierI., Dupuis GirodS.,  (2017). Hematopoietic stem cell transplantation in 29 patients hemizygous for hypomorphic IKBKG/NEMO mutations. Blood
130, 1456–1467.2867973510.1182/blood-2017-03-771600PMC5609334PeledA., SarigO., SunG., SamuelovL., MaC.A., ZhangY., DimaggioT., NelsonC.G., StoneK.D., FreemanA.F.,  (2019). Loss-of-function mutations in caspase recruitment domain-containing protein 14 (CARD14) are associated with a severe variant of atopic dermatitis. J. Allergy Clin. Immunol.
143, 173–181.e10.3024835610.1016/j.jaci.2018.09.002SchwabC., GabryschA., OlbrichP., PatiñoV., WarnatzK., WolffD., HoshinoA., KobayashiM., ImaiK., TakagiM.,  (2018). Phenotype, penetrance, and treatment of 133 cytotoxic T-lymphocyte antigen 4–insufficient subjects. J. Allergy Clin. Immunol.
142, 1932–1946.2972994310.1016/j.jaci.2018.02.055PMC6215742SpinnerM.A., SanchezL.A., HsuA.P., ShawP.A., ZerbeC.S., CalvoK.R., ArthurD.C., GuW., GouldC.M., BrewerC.C.,  (2014). GATA2 deficiency: a protean disorder of hematopoiesis, lymphatics, and immunity. Blood
123, 809–821.2422781610.1182/blood-2013-07-515528PMC3916876TangyeS.G., Al-HerzW., BousfihaA., ChatilaT., Cunningham-RundlesC., EtzioniA., FrancoJ.L., HollandS.M., KleinC., MorioT.,  (2020). Human Inborn Errors of Immunity: 2019 Update on the Classification from the International Union of Immunological Societies Expert Committee. J. Clin. Immunol.
40, 24–64.3195371010.1007/s10875-019-00737-xPMC7082301ToubianaJ., OkadaS., HillerJ., OleastroM., Lagos GomezM., Aldave BecerraJ.C., Ouachée-ChardinM., FouyssacF., GirishaK.M., EtzioniA.,  (2016). Heterozygous STAT1 gain-of-function mutations underlie an unexpectedly broad clinical phenotype. Blood
127, 3154–3164.2711446010.1182/blood-2015-11-679902PMC4920021TownsleyD.M., DumitriuB., and YoungN.S. (2014). Bone marrow failure and the telomeropathies. Blood
124, 2775–2783.2523719810.1182/blood-2014-05-526285PMC4215309

## Figures and Tables

**Table 1.** Patient Characteristics Some patients had multiple samples collected over time at different visits, thus the number of samples can exceed the number of patients indicated.

Condition	Subject Count		Sample Count			Age at Sample Drawn	Sex	Race					
	Primary	Set Aside	Serum Proteomics	CBC + TBNK immune cell phenotyping	Whole Blood Transcriptomics	median [min-max] (Years)	Male	Asian	Black/ African American	Hawaiian/ Pacifier Islander	Multiple Race	White	Unknown
**p47-CGD**	18	4	31	33	32	36.8 [14.9-58.3]	12 (54.5%)	-	4 (18.2%)	-	-	17 (77.3%)	1 (4.5%)
**X-CGD**	23	6	41	51	49	31.3 [7.6-52]	28 (96.6%)	1 (3.4%)	4 (13.8%)	-	1 (3.4%)	22 (75.9%)	1 (3.4%)
**CARD14 DN**	2	0	2	2	1	13.25 [12.4-14.1]	1 (50%)	-	2 (100%)	-	-	-	-
**CTLA4**	4	1	7	8	10	31.6 [18.3-57.9]	4 (80%)	-	-	-	-	5 (100%)	-
**DADA2**	8	2	13	13	13	15.2 [7.4-26.3]	7 (70%)	1 (10%)	-	-	-	8 (80%)	1 (10%)
**FCAS**	6	1	7	7	6	21.2 [2.7-55.8]	3 (42.9%)	-	-	-	-	4 (57.1%)	3 (42.9%)
**FMF**	10	2	12	12	13	53.6 [14.2-77.6]	7 (58.3%)	-	-	-	-	12 (100%)	-
**GATA2**	14	4	19	21	17	41.9 [16.4-81.8]	4 (22.2%)	-	-	-	1 (5.6%)	15 (83.3%)	2 (11.1%)
**HIDS**	4	1	6	6	7	19.4 [10.4-20.4]	2 (40%)	-	-	-	-	5 (100%)	-
**IL-12R**	2	1	3	4	4	21.4 [6.5-43.5]	1 (33.3%)	-	-	-	1 (33.3%)	2 (66.7%)	-
**LAD1**	2	0	3	4	5	30.5 [30.3-38.4]	2 (100%)	-	-	-	-	2 (100%)	-
**Muckle-Wells**	3	1	5	5	5	36.5 [7.9-43.8]	2 (50%)	-	-	-	1 (25%)	3 (75%)	-
**NEMO**	2	1	6	6	7	29.9 [8.9-39.2]	3 (100%)	-	-	-	-	3 (100%)	-
**NEMO carrier**	2	0	2	2	2	24.1 [15.3-32.9]	0 (0%)	-	-	-	-	2 (100%)	-
**PAPA Syndrome**	6	2	14	14	11	29.3 [17.5-60.1]	5 (62.5%)	-	-	1 (12.5%)	1 (12.5%)	6 (75%)	-
**PGM3**	6	1	9	11	10	15.5 [3.9-38.7]	6 (85.7%)	-	-	-	-	7 (100%)	-
**PI3K**	9	2	13	17	15	14.75 [9.4-25.9]	3 (27.3%)	1 (9.1%)	2 (18.2%)	-	-	8 (72.7%)	-
**STAT1 GOF**	15	4	31	34	32	29 [16.7-71.1]	5 (26.3%)	-	1 (5.3%)	-	-	18 (94.7%)	-
**STAT3 DN**	32	8	39	50	44	25.7 [6.2-59.9]	21 (52.5%)	1 (2.5%)	5 (12.5%)	-	-	30 (75%)	4 (10%)
**TERC**	2	0	2	2	2	36.65 [29.3-44]	1 (50%)	-	-	-	-	2 (100%)	-
**TERT**	3	1	5	5	3	53.3 [28.5-59.3]	3 (75%)	-	-	-	-	4 (100%)	-
**TRAPS**	10	3	14	14	13	30.7 [12-67.9]	6 (46.2%)	-	-	-	-	12 (92.3%)	1 (7.7%)
**Healthy**	34	8	42	43	44	33.2 [6.1-67.8]	20 (47.6%)	3 (7.1%)	8 (19%)	-	2 (4.8%)	28 (66.7%)	1 (2.4%)
